# Uridine as a potentiator of aminoglycosides through activation of carbohydrate transporters

**DOI:** 10.1126/sciadv.adw7630

**Published:** 2025-09-05

**Authors:** Manon Lang, Stéphane Renard, Imane El-Meouche, Ariane Amoura, Erick Denamur, Léo Hardy, Julia Bos, Tara Brosschot, Molly A. Ingersoll, Eric Bacqué, Didier Mazel, Zeynep Baharoglu

**Affiliations:** ^1^ Institut Pasteur, Université Paris Cité, CNRS UMR3525, Unité Plasticité du Génome Bactérien, 75015 Paris, France.; ^2^ Sorbonne Université, Collège Doctoral, F-75005 Paris, France.; ^3^ Evotec ID (Lyon) SAS, 69000 Lyon, France.; ^4^ Université Paris Cité, INSERM UMR 1137 IAME, F-75018 Paris, France.; ^5^ Mucosal Inflammation and Immunity Team, Université Paris Cité, CNRS, Inserm, Institut Cochin, and Department of Immunology, Institut Pasteur, Paris, France.; ^6^ Epitranscriptomic and Translational Responses to Antibacterial Stress Team, Expression Génétique Microbienne, CNRS UMR8261, Institut Pasteur, Université Paris Cité, Institut de Biologie Physico-Chimique, Paris, France.

## Abstract

Aminoglycosides (AGs) are broad-spectrum antibiotics effective against Gram-negative bacteria, with uptake dependent on membrane potential. However, the mechanisms of AG entry remain incompletely understood. Here, we identify a previously undescribed uptake pathway via carbohydrate transporters in *E. coli*. By deleting or overexpressing 26 carbohydrate transporters, we found that 18 facilitated AG uptake, a mechanism conserved across several Gram-negative ESKAPEE pathogens. Using fluorescent-labeled AGs and flow cytometry, we quantified differential uptake. To enhance AG efficacy, we screened 198 carbon sources for their ability to induce transporter expression using a *cmtA*-*gfp* fusion. Uridine emerged as a strong inducer of *cmtA* and 12 additional AG-importing transporters. Coadministration of uridine considerably improved AG efficacy against clinical and resistant *E. coli* strains by enhancing drug uptake. This combination also improved outcomes in human blood ex vivo and in a murine urinary tract infection model. Given uridine’s clinical safety, it holds promise as an adjuvant to potentiate AG treatment against multidrug-resistant infections.

## Teaser

Uridine boosts aminoglycoside treatment efficiency against antibiotic-susceptible as well as antibiotic-resistant *E. coli* strains.

## INTRODUCTION

Antibiotics have played a crucial role in modern medicine; however, their use is jeopardized by the emergence of resistance in bacterial populations (*1*), with a prevalence of Gram-negative bacteria as highlighted by the World Health Organization (*2*). Bacterial antimicrobial resistance (AMR) was directly linked with 1.27 million deaths in 2019, with only six pathogens accounting for 95% of these deaths, namely, *Escherichia coli*, *Staphylococcus aureus*, *Klebsiella pneumoniae*, *Acinetobacter baumannii*, and *Pseudomonas aeruginosa* (*3*). Among these, all except *S. aureus* are Gram-negative bacteria. AMR-related fatalities thus surpass the combined death toll of malaria and HIV (*3*), underscoring the urgent need for the development of novel therapies. Common resistance mechanisms developed by bacteria consist of limiting antibiotic entry, increasing efflux, altering the antibiotic, or mutating the target. Modulation of intracellular concentrations of antibiotics is one of the most frequent processes leading to resistance, and the entry of antibiotics into Gram-negative bacteria is one of the major bottlenecks in the research of new antibiotics.

Aminoglycosides (AGs) are a class of broad-spectrum antibiotics that effectively cross the double membrane barriers of Gram-negative bacteria (*4*). They are commonly used in clinical settings to treat infections caused by various Gram-negative pathogens, such as pneumonia, sepsis, and urinary tract infections (UTIs) (*5*). However, AG treatment is associated with adverse effects such as ototoxicity (*6*) and nephrotoxicity (*7*, *8*) after long-term treatments.

AG entry into Gram-negative cells is believed to occur in several steps (*9*, *10*). First, electrostatic interactions between AGs and the outer membrane (*9*, *11*, *12*) lead to membrane disruption and AG entry into the periplasm (*11*). Outer membrane porins are also involved in this initial step (*13*). The second step allows AG diffusion from the periplasm into the cytoplasm and relies on the proton motive force (PMF), which is directly linked with electron transport and respiration (*14*, *15*). Last, AGs reach their target, the ribosome, and impair translation (*16*, *17*). Mistranslated membrane proteins further compromise membrane integrity, leading to additional AG uptake in a third step [for a recent review on AG uptake (*18*)]. Apart from nonspecific absorption caused by membrane damage during the second step of entry, there have been suggestions of active transport of AGs through the inner membrane, but the existence of specific transporters of AGs has been elusive since it was first proposed in 1978 that they could be carried by polyamine transporters (*19*). A recent study showed that amino acid carriers in *E. coli* facilitate the transport of AGs (*20*, *21*). We previously aimed to examine the effects of sublethal doses of AGs on gene expression in *Vibrio cholerae*. Our goal was to potentially uncover pathways and processes involved in responding to AG stress, which might not be apparent when focusing only on resistance to high doses. Our approaches enabled us to identify factors contributing to antibiotic tolerance rather than direct resistance (*22*–*24*). We thus decided to investigate alterations in gene expression caused by AGs in *E. coli*, because *E. coli* is recognized as one of the pathogens of critical concern according to the World Health Organization due to frequent carbapenem resistance of some strains, and AGs are commonly used against this species in clinical practice.

In the present study, transcriptomic analysis revealed a decrease in the expression of sugar transporters when grown with tobramycin at 50% of the minimum inhibitory concentration (MIC). We have also observed similar changes in response to 2% MIC of tobramycin in *V. cholerae* (*25*). This might indicate that the down-regulation of sugar transporters is an adaptive response to AGs, prompting us to consider a broader connection between sugar transporters and AG uptake. We provide evidence for a previously unreported mechanism of AG uptake in Gram-negative bacteria. We identified specific carbohydrate transporters involved in AG uptake in *E. coli* and demonstrated that this uptake can be induced through increasing the number of these transporters and does not require changes in the PMF. We propose that the sugar moiety inherent to AG structure allows them to be recognized as substrates by several carbohydrate transporters in Gram-negative pathogens, enabling AGs to exploit bacterial sugar transport systems. We screened for molecules capable of increasing the expression of these transporters both in rich medium and synthetic human urine and identified uridine as a potentiator of AGs. We report potentiation of AGs’ effect not only in laboratory media but also in human blood as well as in vivo, in a mouse bladder infection model. Uridine supplementation increases the expression of AG transporters and offers a means to reduce effective AG doses in vitro and in vivo and to resensitize resistant strains in a clinical setting or in the context of complicated infections. Uridine potentiation could also limit the emergence of AG-resistant clones, thereby reducing recurrent infections.

## RESULTS

### Tobramycin treatment leads to down-regulation of various carbohydrate transporters

**Fig. 1.**
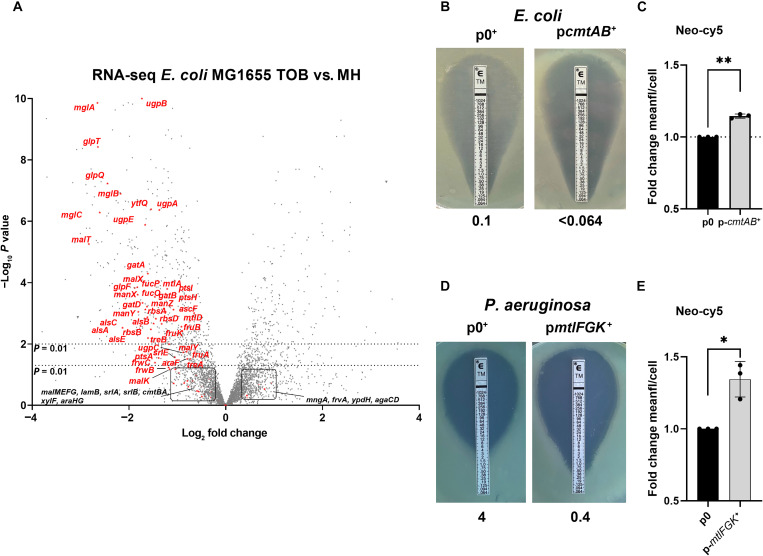
Sugar transporters are involved in tobramycin tolerance. (**A**) Volcano plot comparing transcriptomes of WT *E. coli* K-12 MG1655 grown with and without sub-MIC tobramycin. The *x* axis shows log_2_ fold change expression with tobramycin compared to nontreated condition. The *y* axis indicates negative log_10_ adjusted *P* value. Sugar transporter genes are indicated. (**B** and **C**) CmtA in *E. coli* sensitizes to tobramycin by increasing the uptake. (B) MIC of tobramycin, indicated in micrograms per milliliter, measured using Etests on WT *E. coli* carrying the empty vector (p0^+^) compared to the vector overexpressing CmtA (p*cmtA*^+^). Sodium benzoate (1 mM) as the inducer was added on the medium. (C) Histogram: Uptake of Neo-Cy5 (0.4 μM in rich MOPS) evaluated by flow cytometry on *E. coli* carrying a plasmid overexpressing CmtA compared to the strain carrying the empty vector (p0) (*n* = 6), expressed as fold change of mean fluorescence per cell (compared either to the WT for mutants or to the empty vector for overexpression). Neo-cy5 (0.4 μM) corresponds to neomycin (0.5 μg/ml). A total of 50,000 to 100,000 events were counted for each condition. (**D** and **E**) MtlFGK in *P. aeruginosa* sensitizes to tobramycin by increasing the uptake. (D) Etest on *P. aeruginosa* carrying the empty vector (p0^+^) compared to the vector overexpressing MtlFGK (p*mtlFGK*^+^). Inducer was added to the medium. (E) Neo-Cy5 (0.4 μM) uptake evaluated by flow cytometry on *P. aeruginosa* carrying a plasmid overexpressing MtlFGK (p*mtlFGK*^+^) compared to the strain carrying the empty vector (p0^+^), expressed as fold change of mean fluorescence per cell. A total of 50,000 to 100,000 events were counted for each condition. For statistical significance calculations, we used one-way analysis of variance (ANOVA). *P < 0.05 and **P < 0.01. Number of biological replicates for each experiment: n = 3, unless otherwise indicated. Neo-cy5 uptake was assayed in MOPS Rich. All other experiments were done in MH medium.

To address the effects of AGs on gene expression in *E. coli*, we performed RNA sequencing (RNA-seq) experiments on exponential phase cultures grown with and without tobramycin at 50% of the MIC (Fig. 1A). Gene ontology enrichment analysis identified carbohydrate transport as one of the most down-regulated processes by tobramycin (fig. S1A), together with chemotaxis/locomotion and response to external stimulus. Motility is pleiotropically controlled in *E. coli* (*26*–*28*) and appears to be an adaptation mechanism under diverse stresses including antibiotics (*29*). The large number of carbohydrate transporters identified as involved in an adaptive response to sub-MIC tobramycin caught our attention, because AGs bear a sugar moiety which could potentially be recognized by sugar transporters (tobramycin: 467 Da, gentamicin: 472 Da). The substrate size limit for phosphotransferase system (PTS) carbohydrate transporters is generally up to around 500 Da (*30*), with most substrates being smaller (*31*). ATP synthase binding cassette (ABC) transporters handle substrates with molecular weights up to 1000 Da, depending on the specific transporter (*32*). Down-regulated carbohydrate transporters include (i) PTS transporters MalX (maltose), ManXYZ (mannose), MtlA (mannitol), GatAB (galactitol), FruAB (fructose), SrlE (sorbitol), and TreAB (trehalose) and putative PTS transporters FrwBC, PtsA, and PtsHI; (ii) ABC transporters MalK (maltose), UgpABCE (glycerol), MglABC (galactose), RbsAB (ribose), and AlsABC (allose); and (iii) MFS transporters for glycerol (GlpTQ and GlpF) and fucose (FucP). We decided to further explore the link between AGs and carbohydrate transporters.

### Deletion of single transporters does not strongly affect susceptibility to AGs

We tested whether *E. coli* K12 exhibits phenotypes connecting AG uptake and carbohydrate transporters. First, we generated single deletion mutants of carbohydrate transporters’ transmembrane domains and examined the impact of these deletions on tobramycin susceptibility in an *E. coli* MG1655 background (table S1). Among the 26 deletion mutants tested, only ∆*cmtA* displayed a significant increase in the MIC of tobramycin compared to the wild-type (WT) strain. This was also confirmed in a different genetic background (BW25113; table S1). None of the other transporter deletion mutants showed any impact or only a slight increase in the MIC of tobramycin (table S1). This lack of a pronounced tobramycin resistance phenotype in the single deletion strains may be attributed to compensatory effects among these transporters, some of which share predicted substrates (table S1). Consequently, we decided to investigate the effect of each transport system by overexpressing them in trans.

### Overexpression of 18 different carbohydrate transporters increases susceptibility to AGs

We expressed the *cmtAB* PTS transporter genes from an inducible promoter to evaluate the response to AGs and non-AG antibiotics, as above. Overexpression of the transporter enhanced susceptibility to tobramycin when compared to the empty vector (Fig. 1B and Table 1). This response was specific to AGs, as no effect of the overexpression was observed with non-AG antibiotics (amoxicillin, chloramphenicol, ciprofloxacin, and spectinomycin; Table 1). Subsequently, we cloned 27 selected carbohydrate transporters on the inducible plasmid. These transporters are almost all localized at the inner membrane, except for three outer membrane transporters (*lamB*, *chiP*, and *bglH*)*.* Note that AlsBACE, RbsDABC, AraFGH, AgaCD, and NagE could not be cloned and thus were not tested further. Among the tested 27, 18 demonstrated increased susceptibility to tobramycin and gentamicin compared to the empty vector (fig. S1B and table S1): *cmtAB*, *chbBCA* (chitobiose PTS), *srlEAB* (glucitol PTS), *ascF* (cellobiose PTS), *malEFG* (maltose ABC), *fruBKA* (*fructose PTS*), *frwBC* (fructose-like PTS*)*, *mngA* (fructose-like PTS), *ypdGH* (fructose-like PTS), *bglF* (β-glucoside PTS), *malX* (maltose PTS), *manXYZ* (mannose-PTS), *gatABC* (galactitol PTS), *treB* (trehalose PTS), *bglH* (β-glucoside porin), *glpTQ* (glucose-6-phosphate major facilitator protein), galactose ABC (mglBAC) and *glvCB* (putative arbutine PTS)*.* The phenotype was milder and more variable for *frwBC*. The overexpression of five transporters also sensitize to ciprofloxacin (fig. S1B): *malEFG*, *bglH*, *manXYZ*, *gatABC*, *treB*, and *glpTQ* [already described as a ciprofloxacin transporter (*33*)]. These observations suggest the involvement of multiple carbohydrate transporters in susceptibility to AGs under our experimental conditions.

**Table 1.** MIC (micrograms per milliliter) of different antibiotics. Determined by Etest for all antibiotics except for neomycin, for which Etests are not available (microtiter broth dilution method) in MH medium with1 mM sodium benzoate for gene expression from plasmids. The microtiter broth dilution method (bold) was also used to validate the results obtained for *E. coli*. For *P. aeruginosa*, *OprB* is a transporter for glucose, mannitol, fructose, and glycerol; AstB for mannose and glucose. For both *P. aeruginosa* and *A. baumannii*, FruA is a transporter for fructose. p0, empty plasmid; p-*geneX*, same plasmid overexpressing the indicated transporter gene; TOB, tobramycin; KAN, kanamycin; GEN, gentamicin; AMI, amikacin; STR, streptomycin; SPE, spectinomycin; CIP, ciprofloxacin; TRM, trimethoprim; AMX, amoxicillin; CHL, chloramphenicol.

MIC	AGs					Other families				
	TOB	GEN	AMI	STR	NEO	SPE	CIP	TRIM	AMX	CHL
*E. coli* p0	0.1	0.064	0.38			6	0.008	0.5	6	1.5
*E. coli* WT p-*cmtAB*^+^	<0.064	0.02	0.25			6	0.008	0.5	6	1.5
***E. coli* p0**	**1**	**0.57**	**2.5**	**6**	**12.5**		**0.007**			
***E. coli* WT p-*cmtAB*** ^**+**^	**0.5**	**0.43**	**1.75**	**3**	**6.25**		**0.007**			
*P. aeruginosa* p0	4	4					0.094			16
*P. aeruginosa* p-*oprB*^+^	1.5	2					0.094			16
*P. aeruginosa* p-*gtsB*^+^	0.4	1.5					0.094			16
*P. aeruginosa* p-*mtlFGK*^+^	0.4	1.5					0.094			16
*P. aeruginosa* p-*oprB2*^+^ (PA2291) -*oprB* homolog	1	1					0.094			16
*P. aeruginosa* p-*fruA*^+^	0.4	1.5					0.094			16
*A. baumannii* p0	0.1									
*A. baumannii* p-*fruA*^+^	<0.064									

### Carbohydrate transporters promote AG uptake

To address whether differential expression of carbohydrate transporters modulates AG uptake, we decided to use Neomycin-Cy5 (Neo-Cy5) as previously described (*34*). Neo-Cy5 is a fluorescent AG (neomycin), coupled to the fluorophore Cy5, specifically designed for bacterial uptake studies (*35*). Neo-Cy5 was previously validated to mimic the properties of AGs in terms of uptake, mode of action, and activity against Gram-negative bacteria (*35*). CmtAB overexpression led to increased Neo-Cy5 uptake, indicating an increase in AG uptake (Fig. 1C), confirming that CmtAB is involved in AG uptake. Note that Cy5 can have direct effects on the membrane via hydrophobic perturbation (*36*) and that the conjugated Neo-Cy5 molecule (667 Da) could be too large to be transported by porins such as LamB, predicted to transport molecules of up to 600 Da in size (*30*), and could transport tobramycin (467 Da) and potentially neomycin (614 Da).

We next investigated whether AG uptake through carbohydrate transporters occurs in other Gram-negative pathogens, namely, *P. aeruginosa* and *A. baumannii*. In *P. aeruginosa*, we overexpressed five transporters involved in glucose, mannose, maltose, and fructose uptake*.* This resulted in up to 10-fold increased susceptibility to AGs (tobramycin and gentamicin; Fig. 1D and Table 1) but not to other antibiotic families (Table 1). To confirm increased AG uptake, we measured Neo-cy5 fluorescence in the strain overexpressing *mtlFGK*, demonstrating a 1.3-fold higher uptake of AGs compared to the empty vector after 15 min of sub-MIC treatment (Fig. 1D). For *A. baumannii*, overexpression of the PTS *fruA* also decreased the MIC of tobramycin to <0.064 compared to 0.1 with the empty vector (Table 1). Together, these observations support the hypothesis that multiple sugar transporters play a role in AG uptake and suggest a strategy in which therapeutic efficacy of AGs may be enhanced through increased expression of selected transporters.

### Uridine activates the expression of 14 sugar transporters involved in AG sensitization

With this hypothesis in mind, we set out to identify conditions that induce the expression of selected carbohydrate transporters and subsequently enhance AG uptake. As a proof of concept, we chose to use the CmtAB transporter’s promoter. The regulation of sugar transporters is known to involve CRP (cyclic adenosine monophosphate repressor protein), the primary regulator of carbon catabolic repression (*37*). In this process, the presence of glucose, which is the preferential carbon source in *E. coli*, decreases the expression of nonglucose sugar transporters (*38*). Note that the overexpressions of some transporters did not show increased sensitivity to AGs while regulated by CRP (e.g., MtlA or XylEFG), suggesting that not all CRP-regulated sugar transporters are involved in AG uptake. We constructed a plasmid carrying a transcriptional fusion between the *cmtAB* operon’s promoter and green fluorescent protein (GFP) and confirmed that *cmtAB* is controlled by CRP (fig. S3A). Considering the well-established regulation of sugar transport by carbon catabolite control, we investigated the potential involvement of the CRP regulator and found that CRP contributes to AG susceptibility (fig. S3BC) and uptake (fig. S3D) in *E. coli.* As deletion of *crp* causes growth impairment even in laboratory conditions, we did not consider it for further study.

**Fig. 2.**
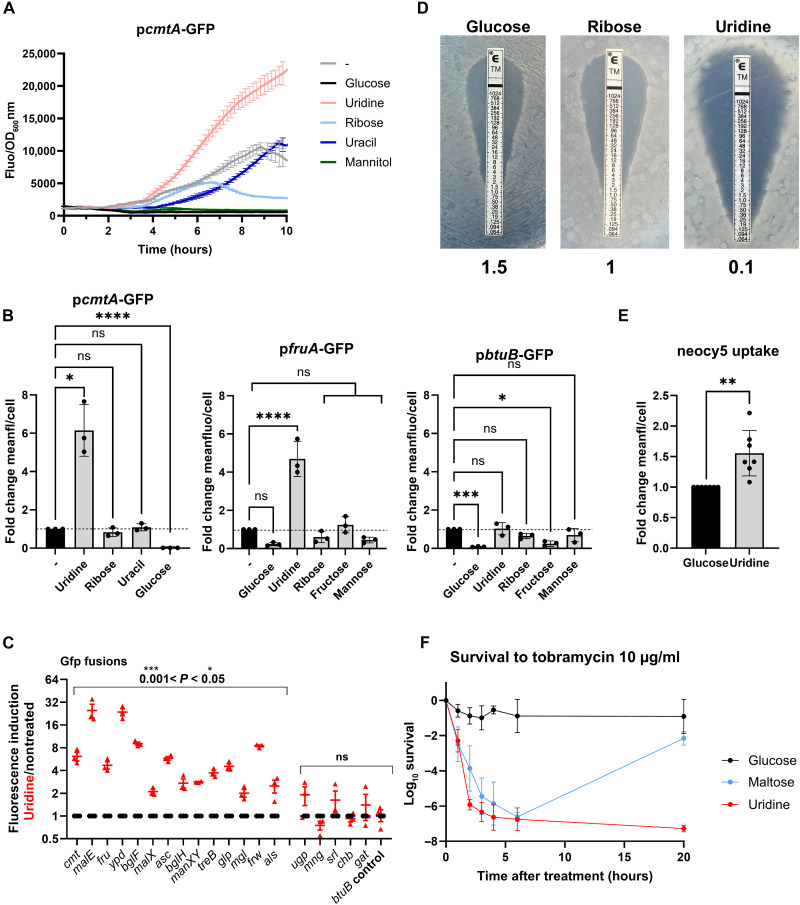
Uridine increases *cmtA* expression and decreases the MIC of tobramycin by enhancing its uptake. (**A**) Growth-normalized GFP expression (Fluo/OD_600_) from the *cmtA* promoter under various conditions (±0.5% glucose, uridine, ribose, and mannitol; ±0.1% uracil). Data show mean ± SD from three biological replicates. (**B**) GFP fluorescence from *cmtA*, *fruB*, or *btuB* promoters quantified by flow cytometry, shown as fold change versus no supplement. Conditions: ±0.5% glucose, uridine, ribose/mannose or 0.1% uracil. Stats: One-way ANOVA, *n* = 3. (**C**) GFP fluorescence from sugar transporter promoters with 0.5% uridine. Mean ± SD from three to six replicates. Stats: Welch’s *t* test. (**D**) Tobramycin MICs (micrograms per milliliter) of WT *E. coli* ±0.5% glucose, ribose, or uridine (Etest). (**E**) Neo-Cy5 (0.4 μM) uptake in WT *E. coli* ±0.5% glucose or uridine, shown as fold change versus glucose (*n* = 7; 50 to 100,000 events per condition). Neo-cy5 (0.4 μM) corresponds to neomycin (0.5 μg/ml). (**F**) Uridine (0.5%) enhances killing and prevents resistance: survival of WT *E. coli* after tobramycin (10 μg/ml) exposure over time ±glucose, maltose, or uridine. Data: Mean ± SD, *n* = 3 unless noted otherwise. Stats: Welch’s *t* test. Media: Neo-Cy5 in MOPS Rich; others in bactotryptone. Significance: ****P < 0.0001, ***P < 0.001, **P < 0.01, *P < 0.05, and *P* > 0.05 [not significant (ns)]*.*

To explore potential carbohydrate sources that could activate the expression of the *cmtA* promoter, we used this fluorescent tool to screen a total of 198 molecules in plates from the Biolog Phenotype Microarray system. We inoculated these plates with WT *E. coli* carrying the P*cmtA*-GFP plasmid and monitored growth by optical density at 600 nm (OD_600_) and GFP fluorescence over a 10-hour period. By calculating the fluorescence-to-growth ratio, we identified uridine as the most potent activator of P*cmtA*-GFP (ratio of 9959) compared to glucose which had one of the lowest levels of *cmtA* expression (ratio of 698) (fig. S4). Uridine was followed by bromo-succinic acid and inosine. We also screened a plate with nucleosides and nucleotides, in which uridine once again showed the highest activation ratio (7601), followed by inosine (6235), guanosine (5669), cytidine (5177), adenosine (5154), thymidine (4152), xanthosine (3907), and lastly glucose as the negative control (ratio of 839) (fig. S4). To validate the findings from the high-throughput Biolog screens, we measured the effect of uridine on fluorescence in standard media, using both a plate reader (Fig. 2A) and flow cytometry (Fig. 2B and fig. S2B). Consistently, a sixfold increase in fluorescence was observed when the media was supplemented with uridine compared to the condition without additional carbohydrates. Uridine consists of a sugar moiety, ribose, and a nucleotide moiety, uracil. The induction of the *cmtA* promoter was not observed with ribose or uracil alone (Fig. 2B).

We next asked whether uridine can activate the promoters of 18 other carbohydrate transporters involved in AG uptake according to overexpression results. Notably, FruA is the only conserved PTS system present in both enterobacteria and *Pseudomonadales*, annotated as a fructose-specific transporter. While the four promoters of *srl*, *mng*, *gat*, *and chb* operons did not respond to uridine, GFP fusions with 14 promoters exhibited increased fluorescence with uridine, including *cmt*, *fru*, *ypd*, *malE*, *bglF*, *malX*, *asc*, *bglH*, *manX*, *tre*, *glp*, *mgl*, *frw*, and *als* (Fig. 2, B and C). To rule out any potential pleiotropic effects of uridine on gene expression under our experimental conditions, we tested the effect of uridine on BtuB, the transporter for vitamin B12. The P*btuB*-GFP construct did not show an increased GFP expression when the medium was supplemented with uridine (Fig. 2B), indicating that not all transporters’ expression responds to uridine. To assess the relative importance of CmtAB in AG uptake, we measured gentamicin MIC with and without uridine under conditions of CmtAB overexpression. Uridine still enhanced gentamicin activity, indicating that other transporters contribute to potentiation and that CmtAB is not the primary transporter involved in AG uptake (table S1). These results suggest that uridine activates transcription of multiple sugar transporters, as summarized in table S2. Note that our data do not rule out other potential effects of uridine on cell physiology that could influence AG activity, as we did not investigate the broader impact of uridine on overall metabolism.

### Uridine supplementation decreases the MIC of AGs by increasing their uptake

As uridine enhanced the expression of carbohydrate/AG transporters, its addition in growth media would be expected to increase bacterial AG uptake, resulting in a lower dose of AGs needed to kill bacteria. Since AGs are bactericidal, this would result in reduced survival (increased killing) to lethal AG treatment and also decreased MIC. We measured MIC values of various antibiotics in *E. coli*, in the presence of different carbohydrate sources (Table 2). In the case of AGs, supplementation with uridine decreased the MIC of tobramycin and gentamicin by 10-fold compared to glucose as a baseline (Fig. 2D and Table 2). No increase in susceptibility was observed to ampicillin or chloramphenicol (Table 2). Unlike with uridine, no significant decrease in MIC was observed upon supplementation with uridine’s sugar moiety, ribose (Fig. 2D and Table 2).

**Table 2.** Effect of different carbohydrates on the MIC (micrograms per milliliter) of different antibiotics. Determined by Etest (unless otherwise indicated), in bactotryptone. “−” means the condition was not tested. Tob, tobramycin; Kan, kanamycin; Gen, gentamicin; Neo, neomycin; Cip, ciprofloxacin; Amp, ampicillin; Chlo, chloramphenicol; AMX, amoxicillin.

	AGs						
Substrate (carbohydrate) supplementation at 0.5%	Tob	Gen	Kan	Neo*	Cip	Chlo	Amp
***E. coli* WT**							
Glucose	1.5	2	2	4*	0.12	1.5	1.5
Uridine	0.1	0.1	0.75	2*	0.06	1.5	1.5
Ribose	1	1	2	−	0.06	1.5	1
Glycerol	0.4	−	−	−	−	−	−
Fructose	1	−	−	−	−	−	−
Galactose	1	−	−	−	−	−	−
Mannitol	1	−	−	−	−	−	−
d-galactosamine	0.3	−	−	−	−	−	−
Mannose	1	−	−	−	−	−	−
Maltose	0.4	−	−	−	−	−	−
Cytidine	0.1	0.1	−	−	−	−	−
Adenosine	0.1	0.15	−	−	−	−	−
Thymidine	0.4	−	−	−	−	−	−
Inosine	1	1.5	−	−	−	−	−
Glucose (MOPS)	−	−	−	1*	−	−	−
Uridine (MOPS)	−	−	−	0.5*	−	−	−
***E. coli *** **Δ** ***crp***							
Glucose	6	−	−	−	−	−	−
Uridine	6	−	−	−	−	−	−
***V. cholerae***							
Glucose	6	−	−	−	−	−	−
Uridine	0.5	−	−	−	−	−	−
***P. aeruginosa***							
Glucose	1	−	−	−	−	−	−
Uridine	0.25	−	−	−	−	−	−
***K. pneumoniae***							
Glucose	1.5	−	−	−	−	−	−
Uridine	0.38	−	−	−	−	−	−
***A. baumannii***							
Glucose	4	−	−	−	−	−	−
Uridine	0.5	−	−	−	−	−	−
***E. coli* UTI89**							
Glucose	2	−	−	−	−	−	−
Uridine	0.4	−	−	−	−	−	−
***E. coli* CFT073**							
Glucose	2	−	−	−	−	−	−
Uridine	0.5	−	−	−	−	−	−

*MIC determined by the microtiter broth dilution method for neomycin for which Etests are not available.

The World Health Organization (WHO) defines ESKAPEE pathogens as bacteria that evade the effects of commonly used antibiotics due to rising multidrug resistance. This group includes *Enterococcus faecium*, *Staphylococcus aureus*, *Klebsiella pneumoniae*, *Acinetobacter baumannii*, *Pseudomonas aeruginosa*, *Enterobacter species*, and *E. coli*. Supplementation with uridine also decreased the MIC of tobramycin compared to glucose for *V. cholerae* (10-fold) and the Gram negative ESKAPEE pathogens *K. pneumoniae* (fourfold), *P. aeruginosa* (fourfold), and *A. baumannii* (eightfold) (Table 2). Thus, the involvement of carbohydrate transporters in AG uptake is a shared characteristic among Gram-negative bacteria, extending beyond a single genus, and the potentiating effects of uridine were present in all the pathogens we examined.

Similar to uridine, supplementation with cytidine, adenosine, and thymidine also resulted in increased susceptibility to AGs (MIC of 0.1 to 0.4 μg/ml) compared to glucose supplementation, whereas inosine did not (MIC of 1 μg/ml) (Table 2). Guanosine was poorly soluble.

We confirmed that uridine increases susceptibility to AGs through increasing AG uptake, using Neo-Cy5 fluorescence per cell, which increased 1.5-fold with uridine after a 15-min sub-MIC tobramycin treatment (Fig. 2E and fig. S2C). In addition, the effect of uridine on AG uptake was not due to changes in membrane potential (fig. S5A) although the membrane potential must be maintained to observe the benefit of uridine, since a CCCP treatment abolishing PMF also abolished a uridine’s potentiating effect (fig. S5B). Microscopy with the pH-sensitive pHrodo dye showed that uridine did not affect intracellular pH (fig. S5, C and D). We also tested and confirmed that uridine-mediated AG susceptibility is not related to uridine catabolism, since deletion of *udk* and *udp* responsible respectively for the degradation of uridine into uracil and ribose-1-phosphate and uridine monophosphate, and overexpression of uridine transporters NupC and NupG (*39*) had no effect on tobramycin tolerance (fig. S6A and table S1), nor did uridine induce the stringent stress response (fig. S6B) (*40*, *41*), block efflux (fig. S6C), or impact membrane permeability (fig. S6, D and E). Last, we detect no impact of uridine on translation-related phenotypes, such as a growth and protein synthesis rate (fig. S7A), RNA modification levels (fig. S7B), or ribosome assembly (fig. S7C). Together, our data support that uridine supplementation leads to a higher AG uptake through increasing the expression of carbohydrate transporters.

### Uridine induces rapid death and limits the selection of AG-resistant mutants

To evaluate the impact of different carbon sources on the effectiveness of tobramycin, we quantified the kinetics of cell death by measuring survival rates during several times points after lethal tobramycin treatment. When glucose (MIC, 1.5 μg/ml; Table 2) was supplemented in the medium, the bactericidal effect of tobramycin was abolished, despite administering the drug at a concentration 10 times higher than the MIC (Fig. 2F). By contrast, supplementation with maltose (MIC, 0.4 μg/ml; Table 2) or uridine (MIC, 0.1 μg/ml; Table 2) enhanced the bactericidal effect of tobramycin to varying degrees (Fig. 2F), with maltose showing the least effectiveness. Notably, in the maltose-treated bacteria, regrowth was observed after 20 hours of treatment. Whole genome sequencing of these colonies pointed to the selection-resistant mutants of *fusA* or *rplL* (table S3), known to be involved in AG resistance mechanisms (*42*, *43*). Conversely, tobramycin showed the highest efficacy on uridine-treated bacteria (Fig. 2F). Uridine supplementation may have prevented the selection of these mutations in the bacterial population. Serial passaging experiments in conditions with and without uridine and sub-MIC tobramycin confirmed that uridine delays the emergence of resistant clones. Resistant colonies appeared after 80 generations in the presence of uridine, compared to only 16 generations in its absence (fig. S8).

### Uridine potentiates AG efficiency *on E. coli* in synthetic urine, plasma, human blood, and UTI

**Fig. 3.**
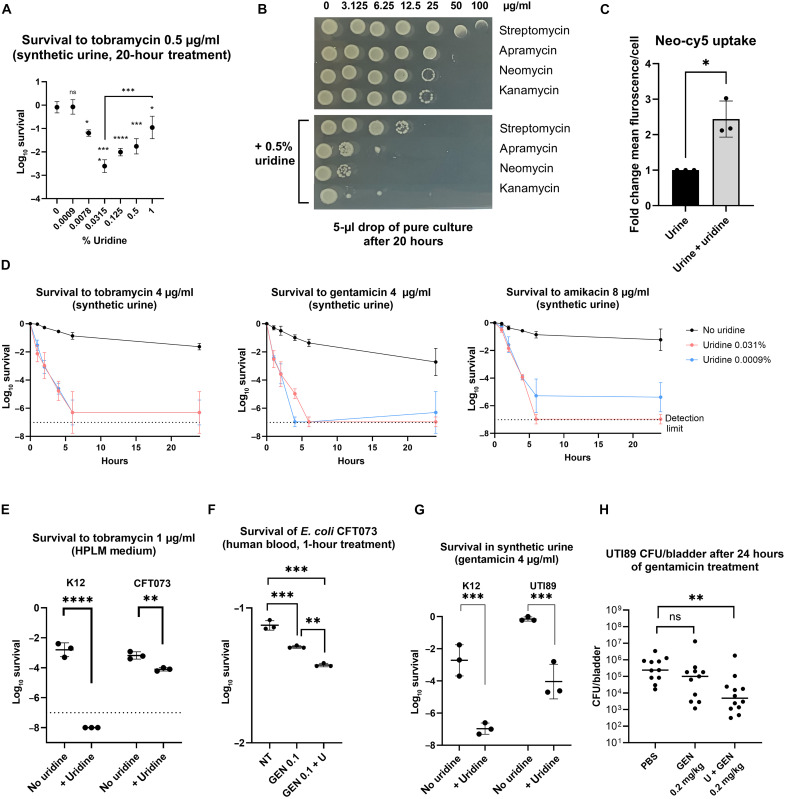
Uridine potentiates AGs in urine and plasma synthetic media by enhancing uptake. (**A**) *E. coli* survival in synthetic urine after 20-hour treatment with tobramycin (0.5 μg/ml) ± increasing concentrations of uridine (%). Significance compares each condition to 0% uridine, except 0.0315% versus 1% as noted (one-way ANOVA, *n* = 3). (**B**) Liquid MIC drop test in synthetic urine with AG (0 to 100 μg/ml) and ±0.5% uridine. Representative of three replicates. (**C**) Neo-Cy5 uptake (0.4 μM) by *E. coli* in synthetic urine ±0.5% uridine, measured via flow cytometry and shown as fold change versus no uridine (*n* = 3; 50,000 to 100,000 events; Welch’s *t* test). Neo-cy5 (0.4 μM) corresponds to neomycin (0.5 μg/ml). (**D**) Survival in synthetic urine with tobramycin (4 μg/ml), gentamicin, or amikacin (8 μg/ml) ± 0.031 or 0.0009% uridine. CFUs measured over 24 hours (geometric mean ± SD, *n* = 3). The dotted line indicates the limit of detection. (**E**) Survival of *E. coli* K12 and CFT073 after 20 hours with tobramycin (1 μg/ml) ± 0.031% uridine in human plasma-like medium (Welch’s *t* test, *n* = 3). (**F**) *E. coli* CFT073 survival after 1-hour treatment with gentamicin (0.1 μg/ml) ± 0.05% uridine (U) in human blood (one-way ANOVA, *n* = 3). NT, non-treated control. (**G**) K12 and UTI89 strain survival in synthetic urine after 20 hours with gentamicin (4 μg/ml) ± 0.031% uridine (Welch’s *t* test, *n* = 3). (**H**) CFUs in homogenized bladders of female C57Bl/6 mice infected with UTI89 and treated with PBS, gentamicin, or gentamicin + uridine (U) for 24 hours. Two pooled experiments, *n* = 5 to 7 per group (one-way ANOVA). For statistical significance calculations, we used one-way ANOVA. Results are two pooled experiments with *n* = 5 to 7 per group. Significance: ****P < 0.0001, ***P < 0.001, **P < 0.01, and *P < 0.05.

UTIs are commonly caused by Gram-negative bacteria and treated using AGs in hospital settings. We tested the effects of uridine on AG susceptibility in synthetic human urine medium (*44*). The MIC of tobramycin in this medium was enhanced 10-fold with addition of 0.5% uridine (1 μg/ml with uridine versus 10 μg/ml without). Next, we compared the effect of different concentrations of uridine, ranging from 0 to 1% in twofold dilutions, on bacterial survival after 20 hours of tobramycin treatment (Fig. 3A). Bacterial cultures with a density of 10^7^ colony-forming units (CFU)/ml were treated with a tobramycin concentration at 50% of the MIC with uridine, i.e., 0.5 μg/ml. We observed the most significant AG potentiation effect with 0.031% uridine. The potentiating effect increased between uridine concentrations of 0.0009 and 0.031% but decreased from 0.031 to 1%. This observation suggests a possible competition between uridine and tobramycin for the transporter responsible for the entry of AGs.

Liquid MIC assays were performed with other AGs, such as streptomycin, neomycin, apramycin, and kanamycin. The addition of uridine decreased the MIC for all four AGs (Fig. 3B). Neo-Cy5 assays demonstrated ~2.5-fold higher uptake at the single-cell level in uridine-supplemented synthetic urine medium, confirming enhanced AG uptake (Fig. 3C and fig. S2C). For cell death kinetic experiments, uridine concentrations of 0.0009% (0.036 mM) and 0.031% (1.27 mM) were used in combination with tobramycin and gentamicin (4 μg/ml), as well as amikacin (8 μg/ml), three commonly used clinical AGs. The addition of uridine resulted in rapid eradication of bacteria, as evidenced by a significant decrease in survival after 24 hours, compared to only a one- to two-log decrease when uridine was absent from the medium (Fig. 3D).

To investigate the potential use of uridine in treating blood infections, e.g., sepsis, we conducted experiments using a plasma-like medium [human plasma–like medium (HPLM)], with or without the addition of 0.031% uridine, the lowest effective concentration. Note that the use of this concentration is also relevant in therapy with intravenous uridine injection in humans (*45*). After 20 hours of treatment, uridine supplementation decreased survival in tobramycin (1 μg/ml) in *E. coli* K12 and CFT073 (*46*) (a pyelonephritis strain isolated from a patient with sepsis) (Fig. 3E). Note that this medium also contains other carbohydrate sources, but they did not interfere with the potentiating action of uridine in this context. K12 appeared to be much more strongly affected by uridine potentiation than CFT073. This may be due to differences in the physiology of the two strains, with CFT073 being a strain adapted to survival in blood. We also confirmed this result using human blood. In these experiments, whole human blood was inoculated with 1.10^4^ CFU of *E. coli* strain CFT073. Gentamicin (0.1 μg/ml) treatment for 1 hour resulted in bacterial survival around 70%, while combination with uridine killed half of the population (Fig. 3F). While the difference is statistically significant, it is relatively small and should be confirmed through in vivo studies or future clinical research.

To determine whether the effect of uridine could also be detected in vivo, we used a mouse UTI model infected with *E. coli* UTI89 (*47*) strain. Among AGs, gentamicin was chosen for this experiment because of its use for UTI infections in the hospital. After confirmation of the efficiency of uridine for potentiating gentamicin on this strain in synthetic urine (Fig. 3G), 6-week-old female C57Bl/6 mice were infected transurethrally with 10^7^ CFU of *E. coli* UTI89 strain and treated 24 hours later with gentamicin (0.2 mg/kg) combined or not with uridine (0.5 g/kg). Bacterial survival was assessed after 24 hours of treatment, i.e., 48 hours postinfection. While gentamicin treatment did not have a significant effect compared to the phosphate-buffered saline (PBS) control, the combination with uridine significantly decreased the amount of bacteria in the bladder (Fig. 3H). Together, these results indicate that uridine constitutes a promising adjuvant to treat UTI and blood infections.

### Uridine potentiation of tobramycin is effective on clinical *E. coli* strains

**Fig. 4.**
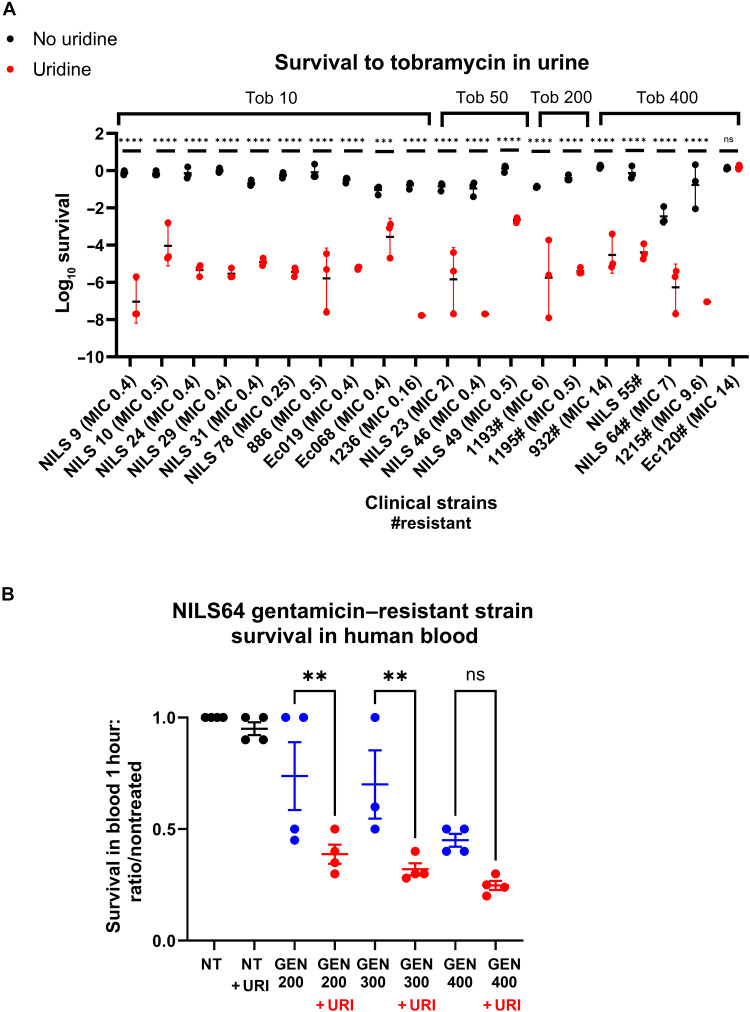
Uridine potentiates AGs on clinical *E. coli* strains A. in synthetic urine. (**A**) Survival of various *E. coli* clinical strains after 20 hours of treatment with 10 (tob 10), 50 (tob 50), 200 (tob 200), or 400 μg/ml (tob 400) of tobramycin, supplemented or not with 0.031% uridine. MICs measured in MH are indicated between parentheses (MIC xx). Survival to tobramycin is shown at the minimal dose for which we could detect an effect of uridine. For statistical significance calculations, we used two way-ANOVA. ****P < 0.0001 and **P < 0.01. Number of independent biological replicates for each experiment: *n* = 3. “#” indicates AG-resistant strains. (**B**) In human blood. Survival of the gentamicin-resistant NILS64 strain to 1-hour gentamicin treatment at indicated concentrations (micrograms per milliliter), supplemented or not with 0.031% uridine. For statistical significance calculations, we used two way-ANOVA. **P < 0.01. Number of independent biological replicates for each experiment: *n* = 4.

Last, we used synthetic urine to assess the AG-potentiating action of uridine on clinical strains of *E. coli*, isolated from a long-stay hospital (see MICs in table S1 and strain descriptions in table S3) or uropathogenic *E. coli* (UPEC) strains from the NILS (natural isolate with low subcultures) collection (*48*), for which the complete genome sequences are available. We first tested strains that do not carry any known tobramycin resistance genes: strain 886 and NILS 9, 10, 23, 24, 29, 31, 47, 49, and 78 with no resistance genes (but different levels of tolerance); strains Ec019 and Ec068 with the MdfA efflux pump associated with a two- to threefold increase in AG MICs (*49*); and strain 1236, which harbors resistance genes against various other antibiotics (β-lactams, sulfonamides, spectinomycin, and streptomycin). In all strains, the addition of uridine (at 0.031%) resulted in strongly decreased survival after 20 hours lethal tobramycin treatment (10 to 50 μg/ml) (Fig. 4A). We also performed checkerboard assays in synthetic urine to assess the interaction between uridine and gentamicin, an AG commonly used to treat UTIs caused by *E. coli* and *K. pneumoniae*. Fractional inhibitory concentration indexes were calculated and indicated a synergistic effect of uridine on gentamicin against the *E. coli* laboratory strain K12, as well as clinical isolates CFT073, NILS23, NILS64, and *K. pneumoniae* (fig. S9).

We also evaluated AG-resistant clinical strains with AG-modifying enzymes (Fig. 4A). With tobramycin concentrations of 200 and 400 μg/ml, which are typically ineffective against these strains, the addition of uridine showed promising results. For strains 1193 and 1195, 0.031% uridine supplementation reduced survival at 200 μg/ml, a concentration achievable in urine. In the case of strain 1215, the potentiating effect of uridine was observed at 400 μg/ml instead of 200 μg/ml. However, for strain Ec120, which carried both efflux pumps and AG modification enzymes, uridine did not demonstrate any effect, even at 400 μg/ml, potentially because of a very high level of resistance (Fig. 4A). Uridine supplementation also potentiates the bactericidal effect of gentamicin on the gentamicin resistant NILS64 strain in human blood (Fig. 4B). In human bladder, after intravenous administration of gentamicin (1 mg/kg), the urinary level of gentamicin reaches concentrations between 113 and 423 μg/ml 1 hour after of treatment and 12 to 271 μg/ml after 2 hours (*50*). Our findings indicate that uridine could thus be used in combination with AGs to effectively treat specific infections caused by resistant *E. coli* strains in clinics.

## DISCUSSION

Modulation of AG uptake into Gram-negative bacterial cells has been primarily associated with changes in PMF (*9*, *14*, *51*), generated by bacterial respiration. While this remains true, our research has identified carbohydrate transporters as players in a different mechanism, which can tune AG uptake in various Gram-negative bacteria. AGs are composed of amine and sugar moieties. Earlier reports proposed that AGs could be transported by amino acid transporters (*20*, *21*), and our results indicate the possibility of direct uptake through sugar transporters, adding to the mechanism of a mere passive uptake through PMF. Previous reports indicated that the efficiency of AG uptake is influenced by carbon sources [such as mannitol (*51*)] and metabolites, through modulation of PMF [e.g., (*51*–*54*)]. Mannitol was found to effectively enhance AG action on persister cells, through stimulation of energy metabolism and thus the PMF. Our microscopy results also suggest that mannitol has an impact on intracellular acidity (fig. S5, C and D). Persister cells better tolerate AGs due to their reduced metabolism, and repowering energy metabolism using mannitol triggers exit from the persister state and increases antibiotic action. Potentiation of AGs on growing cells as we describe in the present study is a different situation than resuscitation of persisters. This mechanism means that sugars, which are capable of regulating the expression of their own transporters, can consequently regulate AG entry through up-regulation of sugar transporters. Note that, although the increase of AG entry through sugar transporters does not require any increase of the PMF, maintaining a membrane potential is necessary, as respiration contributes to adenosine triphosphate (ATP) production, and ATP powers transporters (*55*). Abolishing PMF would also shut down transporters at the membrane. Therefore, the requirement for the presence of PMF in AG uptake could be associated, at least partly, with the activity of the transporters.

The chemical structure of AGs consists of amino sugars linked to an aminocyclitol core structure (*10*). The sugar moiety potentially allows these molecules to be recognized as substrates by bacterial carbohydrate transporters. Consistently, overexpression sugar transporters (such as CmtAB; Table 1) does not affect susceptibility to spectinomycin, which lacks the sugar moiety. There is precedence for antibiotic molecules hijacking bacterial transporters for uptake. For instance, in *P. aeruginosa*, imipenem which has structural similarities to amino acids is transported through the amino acid porin OprD2 (*56*). Our results, however, do not exclude an indirect effect of sugar transporters on AG uptake.

AG resistance is usually associated either with genomic AG-modifying factors, which inactivate the AG molecules, or genetic mutations. These mutations can either affect the target of AGs (the ribosome) or, more frequently, their uptake via decreased PMF (*42*, *57*, *58*). The reason why mutations in carbohydrate transporters have not been previously linked with AG resistance may be explained by the diverse array of transporters capable of transporting AGs. Through systematic overexpression of sugar transporters, we have identified at least 18 transport systems in *E. coli* that are specifically involved in AG uptake. These systems include PTS for various sugars (chitobiose, glucitol, cellobiose/arbutine, fructose and fructose-like, β-glucoside, and maltose) and the ABC transporter MalEFG for maltose. Such redundancy for AG transport is also consistent with the relatively mild phenotypic effects observed in single transporter deletions. The individual impact of sugar transporters on AG susceptibility may appear modest when compared to genetic resistance mechanisms. However, simultaneous activation of multiple transporters can have a substantial effect on sensitization to AGs. We showed that such up-regulation can be achieved by supplementing uridine, the most potent activator of *cmtA* transcription among 188 substrates tested. Uridine potentiated the killing effect of the clinical AGs tobramycin, gentamicin, and amikacin in synthetic urine, a standardized medium that mimics the bacterial growth conditions in UTI. This enhancement was due to an increased uptake of AGs by the bacteria.

Nucleotide metabolism has recently been linked to antibiotic responsiveness (*59*), with an influence on central metabolism. Both purine and pyrimidine biosynthetic pathways have been shown to modulate the inoculum effect in an antibiotic-dependent manner (*60*). Notably, recent findings demonstrate that adenine scarcity enhances the bactericidal activity of antibiotics such as AGs and β-lactams by affecting purine biosynthesis, thereby altering ATP demand, central carbon flux, and the cell’s overall energy balance (*61*). Supplementation with pyrimidines has also been reported to potentiate the effects of ciprofloxacin, ampicillin, and kanamycin by stimulating the tricarboxylic acid (TCA) cycle and respiratory activity, ultimately leading to increased oxidative stress (*62*). Furthermore, in the Gram-positive bacterium *S. aureus*, uracil acts synergistically with gentamicin, again through mechanisms involving enhanced TCA cycle activity, reactive oxygen species generation, and modulation of ATP levels and PMF (*63*). In the present study, however, we observed that uridine’s potentiating effect on AGs does not operate via alterations in PMF or ATP synthesis, indicating a distinct mode of action, involving sugar transporters.

Sugar transport is generally regulated by carbon catabolite control. As predicted (*64*), we showed that CRP serves as a regulator of *cmtA* and contributes to AG susceptibility in *E. coli.* This does not exclude regulation of AG uptake through other transcription factors. *Pseudomonadales* and enterobacteria like *E. coli* (*65*) show differences in catabolic repression and carbon source utilization. Nevertheless, the uptake of AGs through carbohydrate transporters appears to be a shared property among *Pseudomonadales*, as overexpression of carbohydrate transporters also enhanced the killing of *P. aeruginosa* and *A. baumannii* by AGs (Fig. 1D and Table 1). Therefore, improving treatments by exploiting carbohydrate/AG transport represents a promising strategy to combat antibiotic resistance, considering the potential synergistic effects of AG and sugars. Note that although uridine shows a strong efficacy to potentiate AGs, the hierarchy (*66*) of sugar utilization in bacteria implies that the combination with another potentiator [e.g., mannitol (*51*)] would not necessarily have a synergistic effect.

In clinical settings, the emergence of AG resistance has been detected in 5.5% of treated cases (*67*), and inadequate antibiotic dosing has been identified as a primary cause of resistance (*68*). Uridine supplementation, by accelerating the kinetics of bacterial death, may reduce the selection of AG resistance, as we observed in synthetic urine, and potentially limit the occurrence of recurrent infections. Cotreatment of uridine with AGs may be particularly beneficial in the case of UTI but may also be applicable to otitis, or eye infections, where the administered doses can be easily adjusted because of the mode of drug delivery. For pulmonary infections, combining uridine with inhaled tobramycin, for example, could improve outcomes (*69*). Intravenous injection of uridine is already used in clinics to counter the toxicity of fluorouracil during chemotherapy (*70*). Treatment with high doses of uridine (up to 10 g/m^2^) induces no side effects (*45*) in humans and constitutes a promising adjuvant to AG. Moreover, the optimal dose of 0.031% (1.5 mM) of uridine matches the peak of uridine in the plasma (2 mM) observed after uridine injection (*70*). In the bladder, the total excretion is 15 to 40% of the administered dose (*45*, *70*), meaning that a uridine concentration of 0.031% in urine is also possible to achieve. Oral administration of uridine is also possible (*71*).

The World Health Organization has classified AGs as critically important antimicrobials for human medicine (*72*), underscoring the significance of seeking improvements in AG treatments (*73*). The concentrations of AGs used in these experiments were lower than those typically achieved in the bladder during therapy. In humans, the administration of gentamicin (1 mg/kg) leads to urinary concentrations ranging from 113 to 423 μg/ml after 1 hour of treatment and 12 to 271 μg/ml after 2 hours (*50*). Despite this, the addition of uridine still demonstrated a substantial effect, suggesting that the use of uridine for UTI could theoretically be feasible.

The use of uridine as adjuvant to AGs could be a potent approach to enhancing treatment outcomes either by reducing the required AG dosage and mitigating the associated adverse effects or by limiting the appearance of AG resistance and even resensitizing AG-resistant bacteria. Treatment with high doses of uridine (up to 10 g/m^2^) induces no adverse effects in humans (*45*). AGs exhibit a concentration-dependent killing (*74*) and are, thus, more effective in treating bacterial infections when higher doses are administered (*75*). Uridine offers a solution to enhance AG uptake, allowing for increased effective doses in bacteria without escalating toxicity for the patient.

## MATERIALS AND METHODS

### Strains, plasmids, and primers

The strains, plasmids, and primers used in the study are presented in table S3.

### Growth conditions

The use of MH medium is common practice when testing antibiotic resistance/susceptibility. Thus, for MIC determinations and killing assays in the absence of any sugar supplementation, MH was used to determine the MIC of the deletion or overexpression strains and for all the overnight cultures. MOPS Rich (Teknova EZ rich defined medium) was used for experiments involving fluorescence measurements, i.e., for the carbon source screening assay and for the neo-Cy5 assay, to avoid intrinsic fluorescence of other media. For specific carbohydrate substrate susceptibility tests, we used a medium containing 1% bactotryptone and 0.5% NaCl supplemented with the sugar. This medium is traditionally used for studies testing the effect of sugars (e.g., carbon catabolite control) avoiding the presence of other sugars in the medium. To mimic UTIs, the synthetic urine medium was prepared as described (*44*). To mimic human plasma, the HPLM medium (Thermo Fisher Scientific) was used. All bacteria were grown at 37°C with shaking (150 rotations per minute).

### Gene deletions

All engineered *E. coli* strains used in this work are derivatives of *E. coli* MG1655 and were constructed by transduction using P1 stocks prepared from Keio knockouts strains. Kanamycin resistance cassette *aph* was removed using the Flippase Recognition Target recombination (FLP/FRT) system (*76*). Regarding deletion of carbohydrate transporters, for multicomponent PTS systems, we deleted the gene for the membrane-associated component.

### Overexpression of transporters

Overexpression was performed by cloning the genes of interest into the pSEVA-238 vector (*77*) under the dependence of the *Pm* promoter, tested in an MH plate containing 1 mM sodium benzoate as an inducer (*78*), kanamycin for plasmid maintenance and the tested antibiotic (tobramycin, gentamicin, or ciprofloxacin). Experiments have been made in triplicate. One representative plate is presented. The primers and restriction sites used for cloning are listed in table S3.

### MIC evaluation

#### Etest

Overnight cultures in MH were diluted 20× in PBS (except for the Δ*crp* strain and in urine synthetic medium, cultures were not diluted). Then, 300 μl was plated on the appropriate medium: MH for genes deletions; MH supplemented with kanamycin and sodium benzoate for maintenance and induction of the plasmid in overexpression strains; 1% amino acid, 0.5% NaCl, and 0.5% of substrate (e.g., glucose, ribose, and uridine) to assess the impact of carbon sources or in synthetic urine medium with agar. Plates were then dried for 10 min. Etests (Biomerieux) were placed on the plates and incubated overnight at 37°C or until visible growth for synthetic urine medium.

#### Liquid cultures

MICs were determined by the microtiter broth dilution method with an initial inoculum size of 10^6^ CFUs/ml. In MH medium, the MIC was interpreted as the lowest antibiotic concentration preventing visible growth (used for neomycin). In synthetic urine medium, 5 μl of pure culture of each antibiotic dilution was plated, and MIC was interpreted as the lowest concentration preventing growth.

### Neo-Cy5 uptake assay

Quantification of fluorescent neomycin (Neo-Cy5) uptake was performed as described (*34*). Neo-Cy5 is an AG (neomycin) coupled to the fluorophore Cy5 that retained activity and mode of uptake in Gram-negative bacteria (*35*). Overnight cultures were diluted 100× in rich MOPS (Teknova EZ rich defined medium). When the bacterial cultures reached an OD_600_ of 0.25, they were treated with 0.4 μM Cy5-labeled neomycin [equivalent to neomycin (0.25 μg/ml)] for 15 min at 37°C under aluminum foil. In rich MOPS, the MIC of neomycin is 0.5 to 1 μg/ml (Table 2). Thus, the concentration of neo-cy5 used in the uptake experiment is sub-MIC for neomycin. For the assays with different substrates, cultures were washed once with PBS before treatment. Twenty microliters of each treated culture was then used for flow cytometry and diluted in 200 μl of PBS before reading fluorescence. A total of 500 μM tetrachlorosalicylanilide (TCS) (Thermo Fisher Scientific) was used as negative control which reduces PMF and thus AG entry. The measured fluorescence is a sum of periplasmic and cytosolic AG uptake. Flow cytometry experiments were performed as described (*79*). For each experiment, 50,000 to 100,000 events were counted on the Miltenyi MACSquant device with the Y3 laser.

### Evaluation of PMF

Quantification of PMF was performed using the DiBac4 (*3*) [bis-(1,3-dibarbituric acid)-trimethine oxanol] dye that only accumulates in depolarized cells, resulting in an increased fluorescence when the PMF is reduced. Note that the PMF is a function of both the ∆pH gradient and ∆Ψ, and Dibac measures ∆Ψ exclusively. For each sample, 100 μl was treated with Dibac4 (1 μg/ml) (*3*), during 15 min at room temperature under aluminum foil. The *tolC* mutant where indicated (*80*) and 500 μM tetrachlorosalicylanilide TCS (Thermo Fisher Scientific) were used as negative controls. Twenty microliters of the treated culture was then used for flow cytometry and diluted in 200 μl of PBS before reading fluorescence. Flow cytometry was performed with the B1 laser.

### Carbon source screening assay

The fluorescence-activated cell sorting–optimized GFPmut3 (*81*) was fused to promoter of interest and cloned into a plasmid pSC101. For the first screening, overnight cultures of the strain carrying the screening system were diluted 200× in MOPS Rich (Teknova EZ rich defined medium) supplemented with carbenicillin for plasmid maintenance. The phenotype microarray (Biolog) plates PM1 and PM2B (carbon sources) and PM3B for a total of 198 molecules including 7 nucleosides and 6 nucleotides were used for molecule screening. Each well was filled with 100 μl of inoculated media and mixed by pipetting. Media were transferred to 96-well dark-bottom plates (Thermo Fisher Scientific). GFP fluorescence was followed on the Tecan Infinite 200 PRO (Life Science) at 37°C for 10 hours. Fluorescence induction by the substrate was calculated using the ratio fluorescence (t10h-t0h) over growth (t10h-t0h OD_600_).

For flow cytometry quantification, overnight cultures in MH of strain carrying the screening system were diluted 200× in rich MOPS (Teknova EZ rich defined medium) or MH supplemented with carbenicillin for plasmid maintenance and grown overnight, and the molecule was tested at 0.5% (except uracil: 0.1%, limit of solubility). Fluorescence was read on 5 μl of overnight cultures diluted in 200 μl of PBS with the B1 laser. Raw results are accessible on Zenodo public repository (10.5281/zenodo.10805264).

### Stringent response

The P1*rrnB*-GFP fusion was constructed using GFP Ala-Ser-Val degradation tag (ASV) (*24*) and cloned into a plasmid pSC101. This promoter is negatively regulated by a ppGpp (*41*) decrease in fluorescence intensity thus indicates an induction of the stringent response. As controls, tobramycin sub-MIC (50%) treatment (*82*, *83*), stationary phase cultures, and aminotriazole (5 mM) treatment were used, as these conditions are known to trigger the stringent response. Cultures were grown overnight (positive control) or diluted 100× until reaching an OD_600_ of 0.4, in MH medium supplemented with carbenicillin for plasmid maintenance and 0.5% of the mentioned substrate or tobramycin (0.1 μg/ml). Fluorescence was read by flow cytometry, on 20 μl of cultures diluted in 200 μl of PBS.

### Inner membrane permeability assay using propidium iodide

We tested the integrity of the inner membrane using propidium iodide, which accumulates upon inner membrane damage, for example, during polymyxin treatment (*84*). ON culture in MH was diluted 1000× in MH supplemented with the appropriate antibiotic for plasmid maintenance and inducer when needed and grown until an OD_600_ of ~0.3. Then, 1 ml of cultures was treated with propidium iodide (0.02 μg/ml) for 30 min at 37°C under aluminum foil, and polymyxin (1 μg/ml) was used as a control. Twenty microliters of the treated culture was then used for flow cytometry and diluted in 200 μl of PBS before reading fluorescence. For each experiment, 50,000 events were counted on the Miltenyi MACSquant device with the Y2 laser.

### Outer membrane permeability assay using nitrocefin

Nitrocefin [3-(2,4-dinitrostyryl)-(6 R,7 R)-7-(2-thienylacetamido)-ceph-3-em-4-carboxyl acid (Calbiochem)], a chromogenic β-lactamase substrate, was used to assess the outer membrane permeability (*23*, *85*). The WT strain was transformed with the psc101 carrying the *bla* gene, and treated with 0.5% of glucose or uridine overnight, in bactotryptone medium. The WT strain without plasmid (no β-lactamase) was used as negative control. Cells were washed twice with PBS and diluted to 5.10^7^ CFU/ml.

Then, 50 μl of bacteria and 25 μl of the nitrocefin stock solution (0.5 mg/ml) were added to 175 μl of PBS, in 96-well plates. In a plate reader, the OD_490_ was measured every 2 min for 45 min at 37°C, with shaking for 10 s every minute.

### Efflux assay using linezolid

Linezolid is an antibiotic poorly effective against Gram-negative bacteria due to the presence of efflux system, notably AcrAB-TolC (*86*). Bacteria were cultivated for 16 hours in a 96-well plate, with an initial inoculum of 10^6^ CFU/ml, in MH medium, supplemented or not with linezolid (200 μg/ml). Cultures were then plated for colony counting.

### Translation efficiency

GFP translation efficiency was measured by cloning GFP under the inducible Pm promoter in pSEVA-238 (see the “Overexpression of transporters” section). Overnight cultures were diluted 200-fold in tryptone medium containing 1 mM sodium benzoate to induce the Pm promoter and kanamycin to maintain the plasmid, supplemented with glucose or uridine (0.5%), in 96-well plates. Growth and fluorescence were monitored using a plate reader.

### RNA purification and analysis of ribosomal RNA species

Overnight cultures were diluted 1:1000 in MH medium and grown with agitation at 37°C until an OD_600_ of 0.3 (exponential phase). A total of 0.5 ml of these cultures was centrifuged, and the supernatant was removed. Pellets were homogenized by resuspension with 1.5 ml of cold TRIzol reagent. A total of 300 μl of chloroform was added to the samples and mixed by vortexing. Samples were then centrifuged at 4°C for 10 min. The upper (aqueous) phase was transferred to a new 2-ml tube and mixed with 1 volume of 70% ethanol. The homogenate was loaded into an RNeasy Mini kit (QIAGEN) column, and RNA purification proceeded according to the manufacturer’s instructions. Samples were then subjected to deoxyribonuclease treatment using the TURBO DNA-free Kit (Ambion) according to the manufacturer’s instructions. Total RNA samples were then analyzed on an Agilent 2100 Bioanalyzer (Agilent Technologies) using the Agilent RNA 6000 Nano Kit according to the instructions of the manufacturer.

### Quantification of RNA modifications

Indicated modifications, methyl-uridine (m5U, m3U, and Um), pseudouridine (Ψ), methyl-pseudouridine (m1Ψ), dihydrouridine (D), and thio-uridine (S2U and S4U), were quantified using mass spectrometry on RNA extracts from cultures grown in MH with and without 0.5% uridine, as described above. Purified RNA fractions were digested into single nucleosides using the New England BioLabs Nucleoside digestion mix (catalog no. M0649S). Ten microliters of the RNA samples diluted in ultrapure water to 100 ng/μl was mixed with 1 μl of enzyme and 2 μl of Nucleoside Digestion Mix Reaction Buffer (10X) in a final volume of 20 μl in nuclease-free 1.5-ml tubes. Tubes were wrapped with parafilm to prevent evaporation and incubated at 37°C overnight. A mass spectrometer was equipped with an electrospray ionization source (HESI-II probe) coupled with an Ultimate Rapid Separation High-Performance Liquid Chromatography system (3000 RS HPLC) (Thermo Fisher Scientific). The standards were purchased from Epitoire (Singapore).

### Killing assay and persistence

On bactotryptone medium, overnight cultures were diluted 1000× in 25 ml of medium containing 1% bactotryptone and 0.5% NaCl, supplemented or not with glucose, maltose, or 0.5% uridine. Cultures grew to an OD_600_ of 0.3 to 0.4, and a 5-ml aliquot was treated with a lethal concentration of tobramycin (10 μg/ml). Cultures were plated at 0 (t0), 1, 2, 4, 6, and 20 hours after treatment, and survival was calculated by counting CFUs per milliliter after treatment divided by the initial number of CFUs per milliliter (t0). For persistence plot (fig. S8B), only time point 20 hours is shown.

On synthetic urine medium, overnight cultures in MH were diluted 10-fold in PBS. Approximately 10^7^ CFUs/ml were diluted in 200 μl of urine synthetic medium containing or not uridine and treated with AGs, in a 96-well plate, and then incubated at 37°C with 100 rpm. Cultures were plated at 0 (t0), 1, 2, 4, 6, and 24 hours after treatment, and survival was calculated by counting CFUs per milliliter after treatment divided by the initial number of CFUs per milliliter (t0).

Regrowth was observed in the maltose-treated bacteria after 20 hours of treatment. We hypothesized that this regrowth could be attributed to the selection of resistant mutants growing after 6 hours of treatment, as the number of surviving bacteria after 20 hours in the presence of tobramycin was higher than at 6 hours of treatment. To investigate further, we sequenced the genomic DNA from 10 colonies which exhibited a MIC around 1 μg/ml. We identified mutations in the *fusA* gene, which encodes elongation factor G, and the *rplL* gene, which encodes 50*S* ribosomal protein L7/L12.

### Dose-response assay

Twofold dilutions of uridine in urine synthetic medium were prepared into column of a 96-well plate, from 2 to 0.0018%. The first column was only filled with synthetic urine medium without uridine. Then, 100 μl of a solution containing 2× concentration of the tested antibiotic was added, thus diluted uridine and antibiotics by two and increasing the total volume per well at 200 μl. The last line was only filled with urine synthetic medium without antibiotic. Each well was then inoculated with approximately 10^7^ CFUs from MH stationary phase culture and incubated 20 hours at 37°C with agitation (110 rpm). CFUs were counted by plating before and after treatment on MH medium.

### Whole genome sequencing

Genomic DNA was extracted from 500 μl of overnight cultures in MH, using the Blood and Tissue Extraction Kit (QIAGEN) according to the manufacturer’s instructions. The presence of variants (single-nucleotide polymorphism) was analyzed with SnpEff 5.0 (*87*).

### RNA sequencing

Cultures of *E. coli* were diluted 1000× and grown in triplicate in MH supplemented or not with tobramycin (0.1 μg/ml), corresponding to 25% of the MIC in liquid cultures to an OD_600_ of 0.4.

RNAs were purified with the RNeasy mini kit (QIAGEN) according to manufacturer’s instructions. Briefly, 4 ml of RNA-protect (QIAGEN) reagent was added on 2 ml of bacterial cultures for 5 min. After centrifugation, the pellets were conserved at −80°C until extraction. Protocol 2 of the RNA-protect Bacteria Reagent Handbook was performed, with addition of a proteinase K digestion step, such as described in the protocol 4. Quality of RNA was controlled using the Bioanalyzer. Library preparation, sequencing, and analysis were performed as described (*88*). Libraries used were as follows: Illumina Stranded Total RNA Prep and Ligation with Ribo-Zero Plus; sequencing: quality control: 1× iSeq100; NextSeq 2000 P1 100 production (target, 50 M reads SE116 per sample); analysis: quality control (QC), differential RNA, and enrichment.

### Human blood infection

For the testing of the gentamicin sensitive CFT073 strain isolated from urine and blood of a patient with acute pyelonephritis, the study was carried out by QIMA Life Science (1 bis rue des plantes 86160 GENCAY–France). Blood came from a 39-year-old male donor. *E. coli* CFT073 (American Type Culture Collection 700928) was grown overnight in MH and then diluted to an OD_600_ of 1 in PBS. Blood (500 μl) was inoculated with 2.10^4^ CFU/ml (2×) and treated with uridine (0.05%), gentamicin (0.1 μg/ml), or the combination (500 μl; prepared in blood, 2×). Blood containing or not uridine and/or *E. coli* bacteria was then incubated for 1 hour at 37°C under agitation (150 rpm). The 1-hour incubation allows for the evaluation of the treatment effects. Extended incubation in fresh whole blood, which includes both erythrocytes and leukocytes, leads to bacterial killing independent of antibiotic exposure. Bacterial enumeration was carried out before (t0) and after culture on the treated blood of each condition. For each condition, treated and untreated blood was plated onto two MH agar plates [100 μl and the pellet of the remaining volume of blood (900 μl)]. For the testing of the gentamicin resistant NILS64 strain, human peripheral blood sample was collected from a healthy volunteer through the ICAReB-Clin (Clinical Investigation platform) of the Institute Pasteur. The participant received oral and written information about the research and gave written informed consent in the frame of the healthy volunteers COSIPOP cohort after approval of the CPP Est II Ethics Committee (20 February 2023). The same protocol as above was used except that the blood volume was 100 μl, and gentamicin was used at 200/300 or 400 μg/ml.

### UTI model

UTI was induced in 6- to 7-week-old female C57BL/6J mice from Charles River, France as previously described (*47*). Briefly, the human UPEC cystitis isolate UTI89, engineered to express the red fluorescent protein (RFP) and antibiotic resistance to kanamycin (UPEC-RFP) (*47*), was grown statically in LB broth for 18 hours at 37°C in the presence of kanamycin (50 μg/ml). Cultures were adjusted to 2 × 10^8^ CFU/ml in PBS, and 50 μl (10^7^ CFU per mouse) was delivered via catheter directly into the bladder of mice anesthetized by intraperitoneal injection of ketamine (100 mg/kg) and xylazine (5 mg/kg). At 24 hours postinfection, mice were treated with 100 μl of PBS, gentamicin (0.2 mg/kg), or gentamicin (0.2 mg/kg) + uridine (0.5 g/kg) by retro-orbital intravenous injection.

The gentamicin dose was determined as follows: Effective concentrations in UTI models have been reported to be in the range of 1 to 2 mg/kg in various studies (*51*, *89*). Since in vitro studies show that uridine potentiates gentamicin activity by ~10-fold in synthetic media, we selected a gentamicin dose that was 10 times lower, at 0.2 mg/kg.

The uridine dose was determined as follows: Tolerated concentrations go up to 0.5 g/kg in rodents (rats), while 3.5 g/kg causes severe hypothermia (*90*). The excretion rate into the bladder is 7% in rabbits (*91*) and mice (*92*). We need uridine concentration in the bladder to be between 0.03 and 0.5%. We estimate the mouse bladder volume as 0.15 ml. To get 0.5% (5 mg/ml) solution in 0.15 ml, we need 5 × 0.15 = 0.75 mg of uridine. If only 7% of the injected dose ends up in the bladder, total uridine that needs to be injected in the mouse is 0.75 mg/0.07 = 10.71 mg. If a standard mouse weighs 25 g, then we should inject 10.71 mg/0.025 kg = 0.43 g/kg. We thus conducted experiments using uridine (0.5 g/kg), which was well tolerated by the mice. These mice were placed on a heat pad during recovery (under the cage, at 35°C, for ~30 min).

Mice were euthanized at 48 hours postinfection (24 hours posttreatment) by cervical dislocation after isoflurane inhalation. To calculate CFU, bladders were aseptically removed and homogenized in 1 ml of PBS. Serial dilutions were plated on LB agar plates with kanamycin. All animals used in this study had free access to standard laboratory chow and water at all times. Infections were conducted at Institut Cochin in accordance with approval of Autorisation Préfectorale d’Expérimentation Animale – Information Système: APAFIS #34290 by SC3–CEEA34–Université de Paris Cité, at Institut Cochin, in application of the European Directive 2010/63 EU.

### Passaging experiment for the evaluation of resistance emergence

WT *E. coli* K-12 MG1655 cultures were passaged for 80 generations in bactotryptone medium with or without sub-MIC tobramycin or uridine as indicated. The initial MIC of tobramycinin bactotryptone was 1 μg/ml. The initial sub-MIC tobramycin concentration was 0.5 μg/ml (50% MIC) and gradually increased to 1 and 2 μg/ml as indicated on the plot. Overnight cultures were diluted 100× in 25 ml of medium containing 1% bactotryptone and 0.5% NaCl, supplemented or not with 0.5% uridine ± tobramycin. Each condition was tested in biological triplicates. Resistant clones were quantified by plating with a lethal concentration of tobramycin (10 μg/ml).

### Statistical analysis

Means and SDs for growth curves and survival rate and means and geometric means for logarithmic values are presented. For in vitro experiments, an *F* test was first performed to determine whether the variances were equal or different between conditions. For conditions with significantly different variances, Welch correction was applied. For two-groups comparison, a Student’s *t* test was used. One-way analysis of variance (ANOVA) or two-way ANOVA was used for multiple comparisons: ****P < 0.0001, ***P < 0.001, **P < 0.01, and *P < 0.05. The number of replicates for each experiment was 3 < *n* < 7. Experiments were carried out using independent biological replicates (at least triplicates). For in vivo UTI experiments, the nonparametric Kruskal-Wallis test for unpaired data was applied with *P* values corrected for multiple testing using Dunn’s multiple comparisons test. The number of replicates for each experiment was *n* = 5 to 7, and the experiment was repeated twice.
