# Supplementary material for: Uridine as a potentiator of aminoglycosides through activation of carbohydrate transporters
Source: Sci Adv. 2025 Sep 5;11(36):eadw7630. doi: 10.1126/sciadv.adw7630 (PMC12412646; doi:10.1126/sciadv.adw7630)
Supplement: Supplementary file 1 — Figs. S1 to S9 Tables S1 to S3 [file sciadv.adw7630_sm.pdf]

Science Advances
AAAS

Supplementary Materials for

Uridine as a potentiator of aminoglycosides through activation of carbohydrate transporters

Manon Lang *et al.*

Corresponding author: Didier Mazel, mazel@pasteur.fr; Zeynep Baharoglu, zeynep.baharoglu@pasteur.fr

Sci. Adv. **11**, eadw7630 (2025)
DOI: 10.1126/sciadv.adw7630

This PDF file includes:

Figs. S1 to S9
Tables S1 to S3

Figure S1

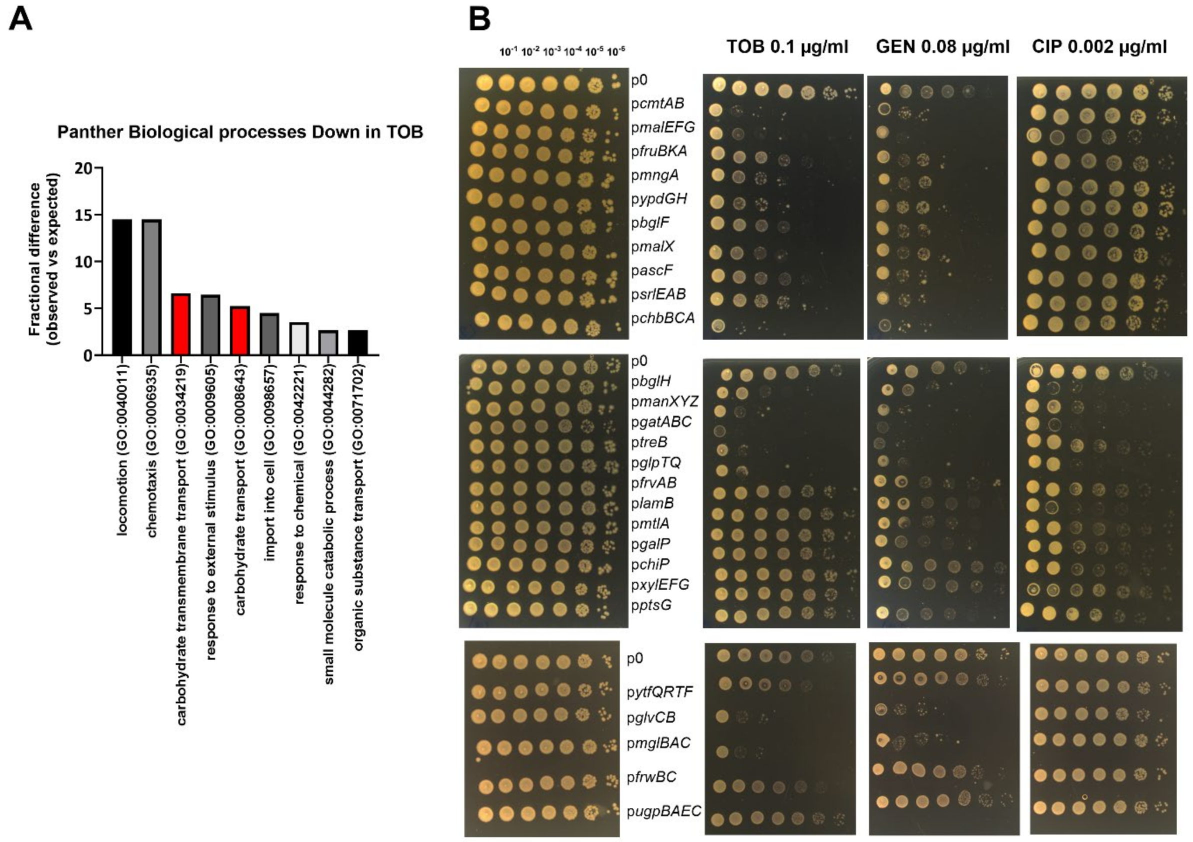

Figure S1. A. Panther biological processes enrichment analysis of transcriptomic study presented in figure 1A. PANTHER GO-Slim Biological Process. Analysis Type: PANTHER Overrepresentation Test (Released 20240226). PANTHER version 18.0 Released 2023-08-01. Analyzed List: 260 genes down in TOB with $p<0.05$. Reference List: *Escherichia coli* (all 4401 genes in database). Test Type: FISHER. Correction: FDR. **B.** Serial dilution (10-fold) of *E. coli* WT overnight cultures carrying plasmids overexpressing sugar transporters (or empty plasmid: p0), in the presence or not of tobramycin 0.1 µg/ml (TOB), gentamicin 0.08 µg/ml (GEN), or ciprofloxacin 0.02 µg/ml (CIP). Experiments were performed in MH, at least three times (independent biological replicates). For overexpression experiments, the media contained kanamycin for plasmid maintenance and sodium benzoate 1 mM as inducer. For C and D, a representative image of 3 independent biological replicates is shown.

Figure S2

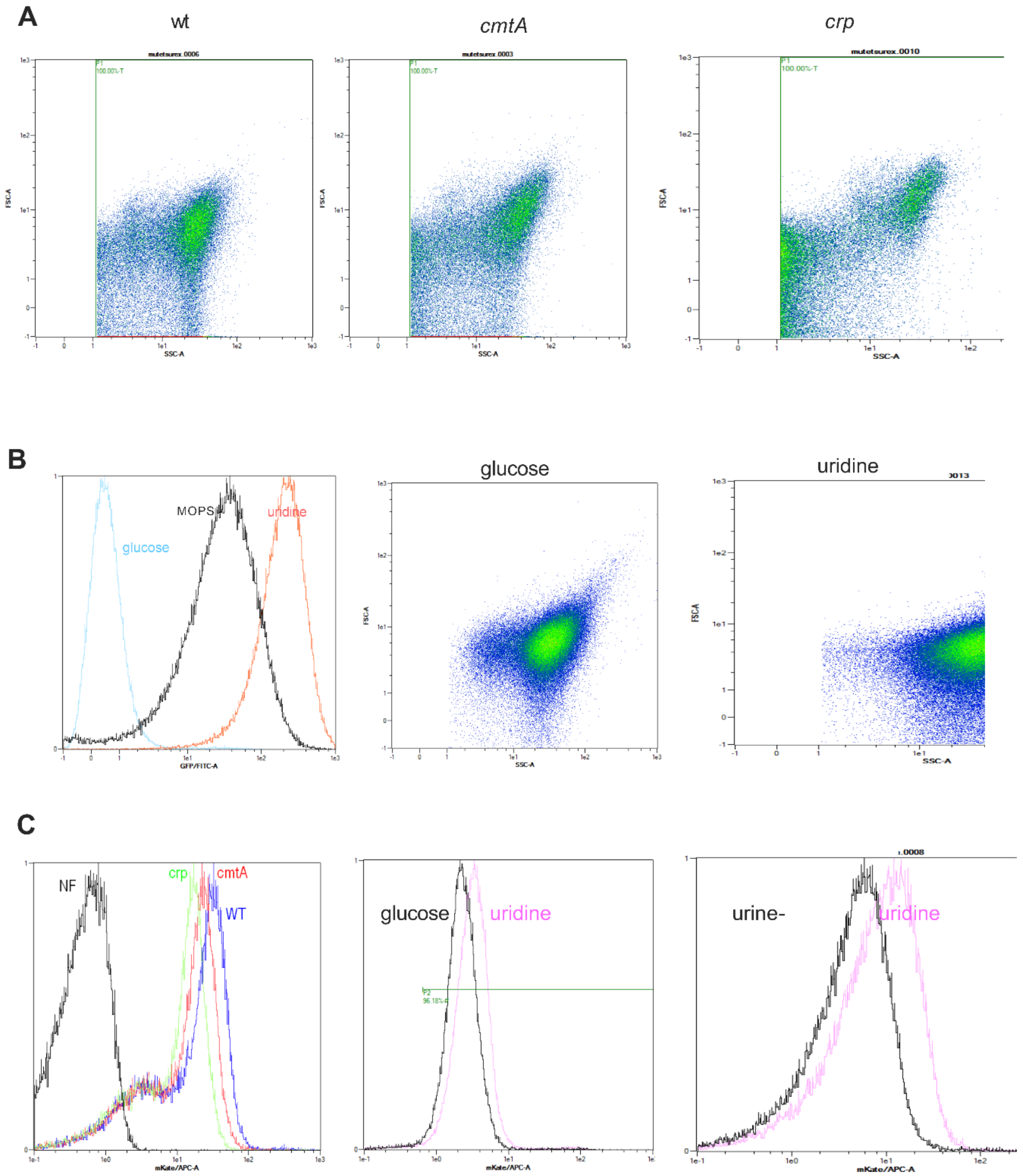

Figure S2: Flow cytometry data. A. Forward Scatter and Side Scatter (size and granularity) of *E. coli* WT, *cmtA* and *crp*. **B**. Forward Scatter and Side Scatter (size and granularity) of *E. coli* growing in MOPS Rich supplemented with glucose or uridine. Associated fluorescence (laser B2) of P*cmtA*-GFP with glucose, uridine or no supplementation. **C.** Fluorescence of Neo-Cy5 detected by the Y3 laser in the mutant and with uridine compared to glucose or in synthetic urine.

Figure S3

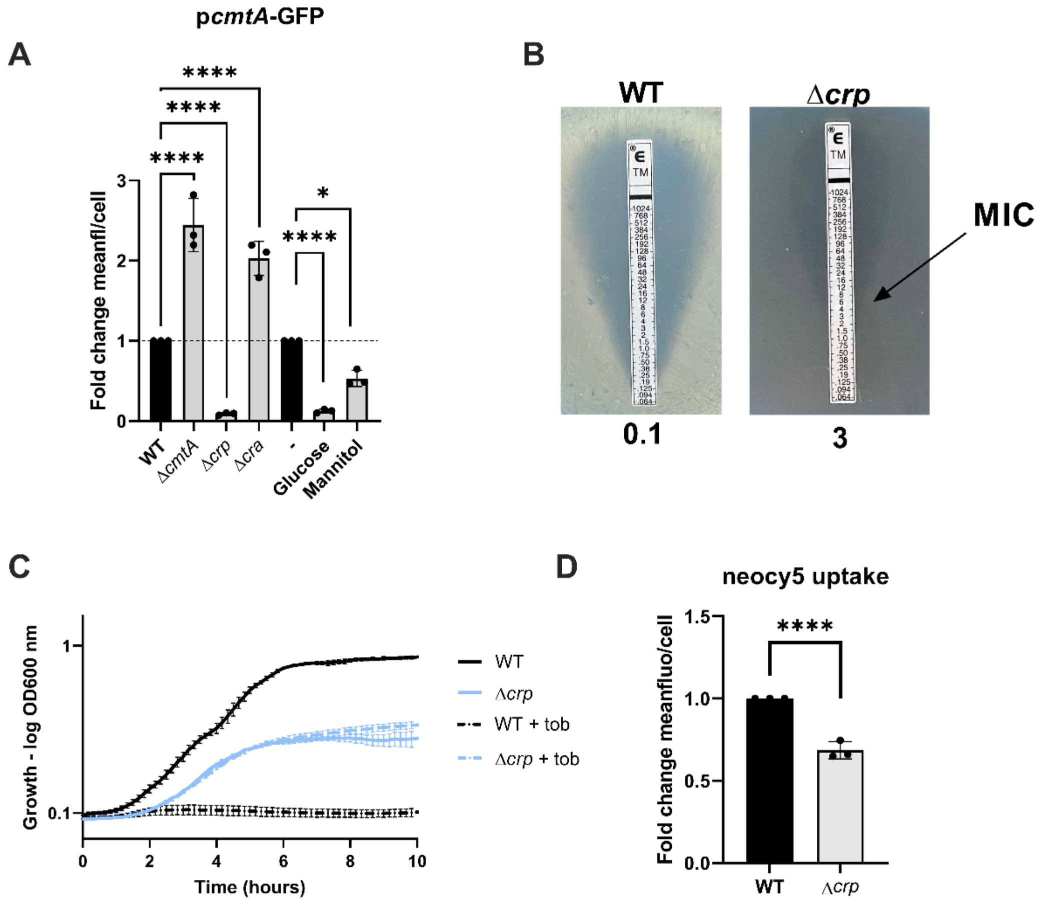

Figure S3. CRP is involved in regulation of *cmtAB* and AG uptake. CRP (cyclic AMP Repressor Protein), a key regulator of carbon catabolite repression, activates transcription of non-preferential sugar transporters. **A**. cmtA promoter activity, measured by GFP fluorescence via flow cytometry, was significantly reduced in *Δcrp* and *ΔcmtA* strains and varied with sugar supplementation (0.5% glucose or mannitol). Data are shown as fold change vs. WT without sugar (one-way ANOVA, n=3; ****p<0.0001, *p<0.05). **B.** Tobramycin MICs (Etest in MH) were 10-fold higher in *Δcrp* compared to WT, despite impaired growth. **C.** Growth curves in MH ± 0.4 µg/ml tobramycin showed Δcrp was resistant to treatment but grew poorly overall (n=3). These data suggest CRP regulates transporters mediating AG uptake. **D**. Neo-Cy5 uptake (0.4 µM = 0.5 µg/ml neomycin) was assessed by flow cytometry in MOPS-rich medium. *Δcrp* showed significantly reduced uptake vs. WT, consistent with the increased MIC (one-way ANOVA, n=3; ****p<0.0001; 50 000–100 000 events per condition). To test if uridine's potentiating effect is CRP-dependent, we measured tobramycin MICs in *Δcrp* with 0.5% glucose or uridine. MIC increased to 6 µg/ml with glucose and remained unchanged with uridine, indicating uridine's effect requires CRP. These findings support a model in which CRP controls AG uptake via activation of non-preferential carbohydrate transporters, including those upregulated by uridine (see Table 2).

Figure S4

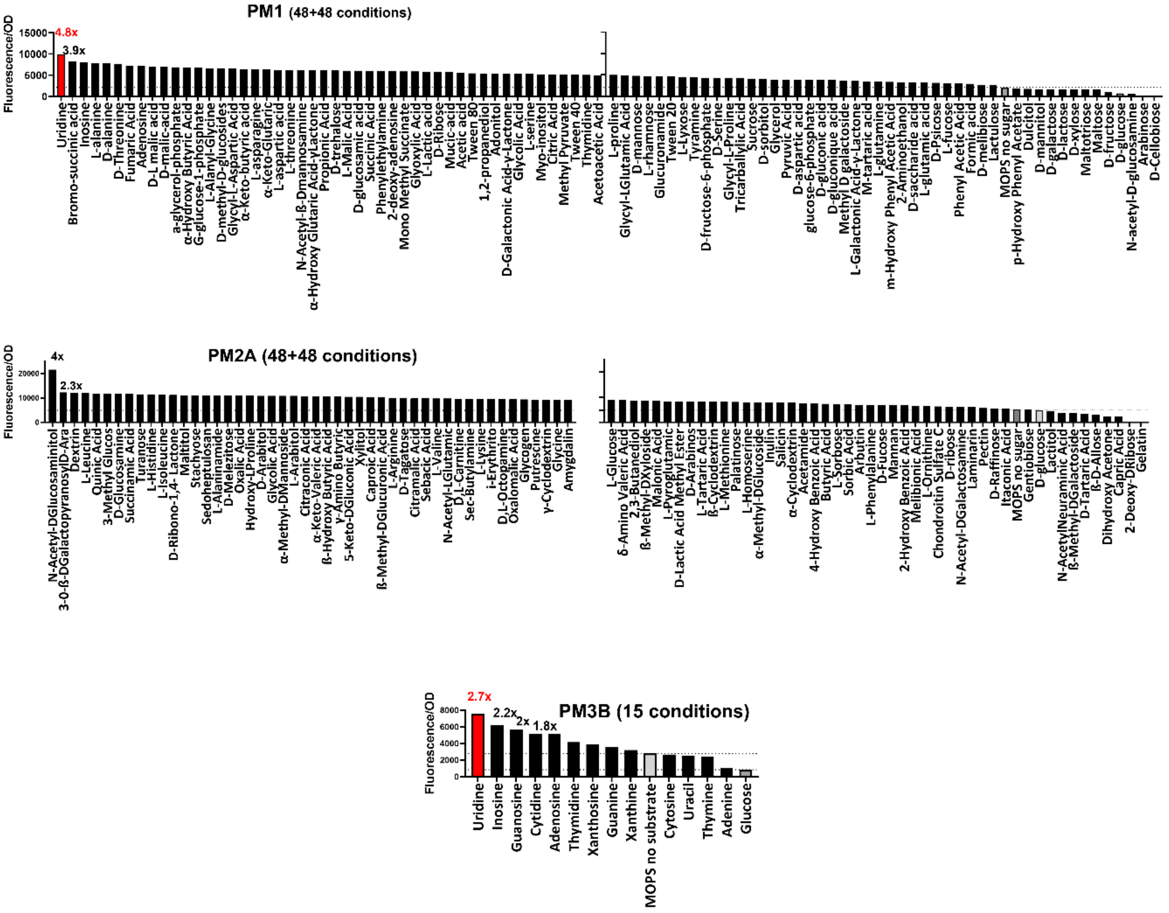

Figure S4. Determination of molecules inducing *PcmtA-gfp* in *E. coli* K-12 in Biolog plates. Ratio of fluorescence over OD 60 nm is shown for the 10-hour time point with each molecule. The FACS optimized GFPmut3 69 was fused to promoter of interest and cloned into a plasmid pSC101. For the first screening, overnight cultures of the strain carrying the screening system were diluted 200X in MOPS Rich (Teknova EZ rich defined medium) supplemented with carbenicillin for plasmid maintenance. The phenotype Microarray (Biolog) plates PM1 and PM2B (carbon sources), and PM3B, for a total of 198 molecules including 7 nucleosides and 6 nucleotides were used for molecules screening. Each well was filled with 100 µl of inoculated media and mixed by pipetting. Media were transferred to 96 well dark-bottom plates (Thermo Scientific). GFP fluorescence was followed on the Tecan Infinite 200 PRO (Life Science) at 37°C for 8 hours. Fluorescence induction by the substrate was calculated using the ratio fluorescence (t8h-t0h) over growth (t8h-t0h OD600nm). Raw results are accessible on Zenodo public repository. 10.5281/zenodo.10805264

Figure S5

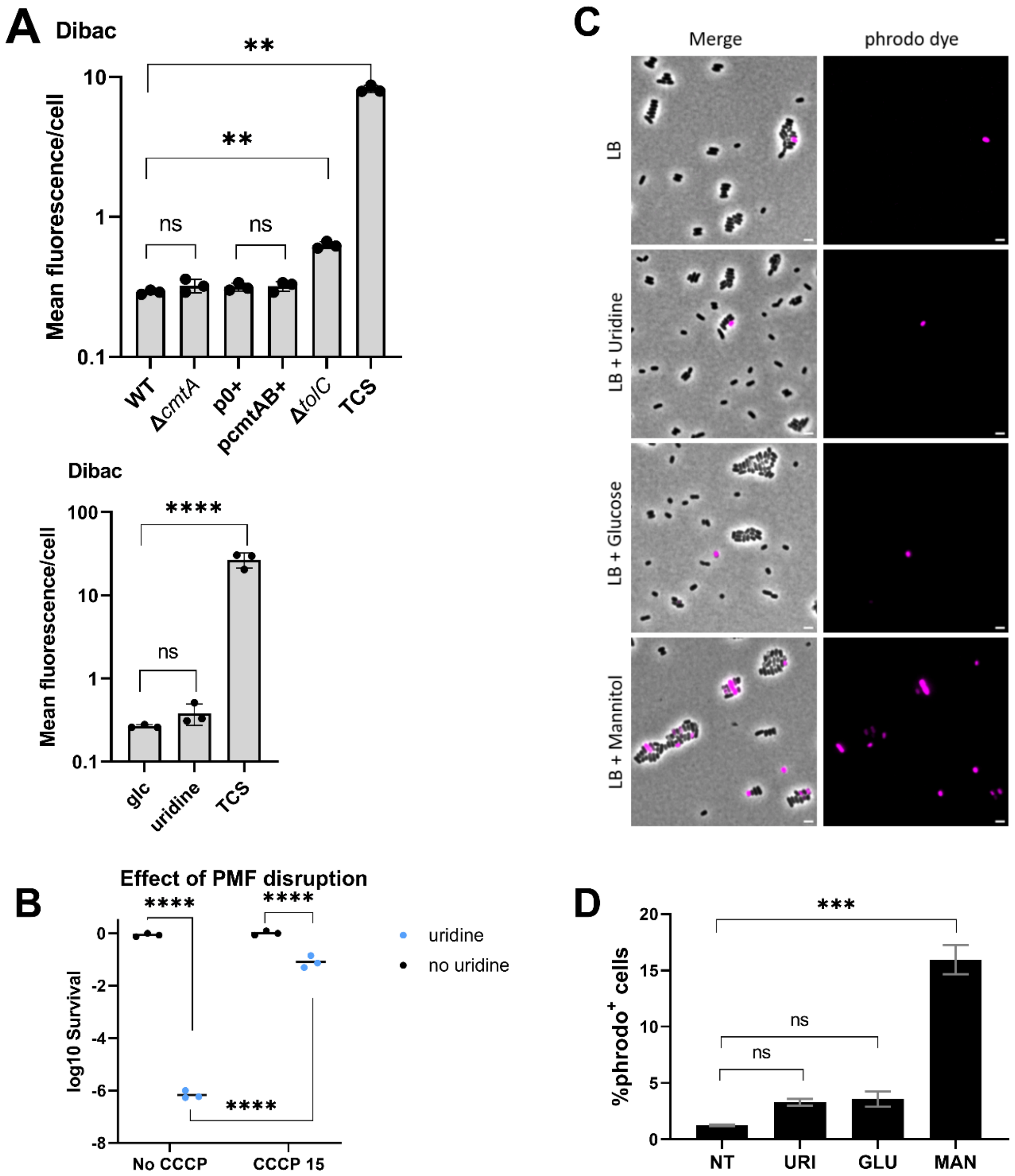

Figure S5: A. No effect of uridine or *cmtAB* overexpression on PMF. PMF was measured using Dibac fluorescence by flow cytometry in *E. coli* WT*, ΔcmtA*, and strains overexpressing CmtAB, compared to an empty vector control (p0). *E. coli* WT was grown in bactotryptone with glucose or 0.5% uridine supplementation. Y-axis shows fold change of mean fluorescence per cell. The *ΔtolC* strain and TCS treatment were used as PMF reduction controls. Biological replicates: n=3. **B. The effect of uridine requires functional PMF.** The effect of uridine requires functional PMF. Survival of *E. coli* to 4 µg/ml tobramycin in synthetic urine with or without 0.5% uridine, in the presence or absence of 15 µM CCCP (protonophore). CFUs were counted after 16 hours of treatment. Fisher's LSD test was used for statistical comparison. Biological replicates: n=3. **C. Quantification of intracellular pH change in the presence of Uridine, Glucose and Mannitol**. (A) Microscopy images of *E. coli* MG1655 grown under the same conditions of MIC tests (Table 2) in the presence of the various sugars. The pH-sensitive dye (pHrodo RED, Thermofischer Scientific #P36600) was added to the cultures 15 min before imaging. Excess of the dye was removed by centrifugation and the cell pellet was resuspended in PBS 1X. Bacteria were immobilized onto an agarose pad and imaged using a widefield microscope (Zeiss) equipped with a Plan Apo 63× objective (+optovar

1.6×), and a Hamamatsu sCMOS ORCA-Flash 4.0 v3. Images shown are a Merge of Phase contrast and DsRed channels, and DsRed channel (pHrodo+ cells indicate a pH decrease from neutral to acidic). The scale bar is 2 microns in all images. **D**. Quantification of microscopy images. Total cell count: n=964 (NT: non-treated), n=1397 (Uri: uridine), n=1211 (Glu: glucose), n=926 (Man: mannitol). n=3**.** Standard error is shown.

Figure S6

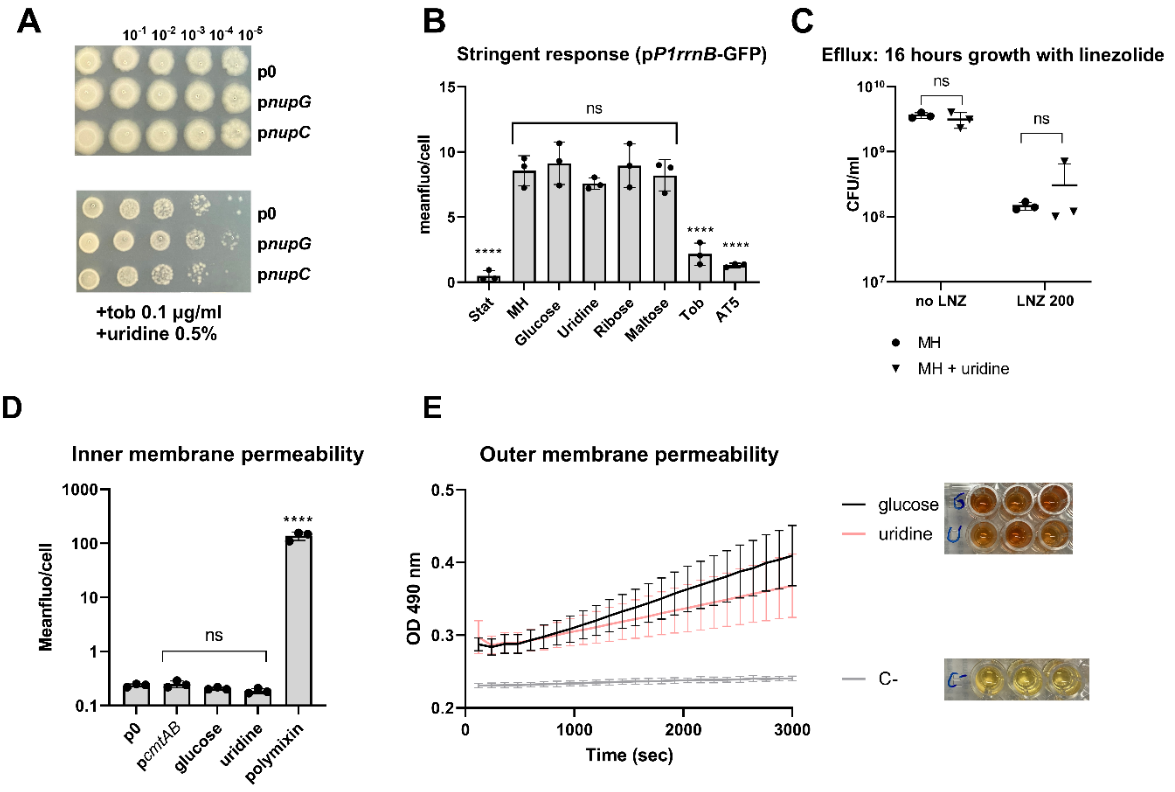

Figure S6: Uridine mediated AG susceptibility is not related to uridine catabolism or stress responses. A. *E. coli* carrying the empty plasmid (p0) or plasmids overexpressing NupG or NupC (uridine transporters) were tested for susceptibility to tobramycin. Cultures were grown overnight in MH, and susceptibility was assessed by serial dilutions and spotting on plates with or without 0.1 µg/ml tobramycin and 0.5% uridine. **B.** GFP fluorescence from the P1rrnB promoter was quantified in exponential phase using flow cytometry, depending on the added substrate (glucose, uridine, ribose, or maltose 0.5%) in MH media. Stationary phase cultures, cells treated with sub-MIC tobramycin (0.06 µg/ml), or aminotriazole were used as positive controls for stringent response activation as previously shown (10.1093/femsml/uqac019, 10.1007/BF00339584; 10.1128/jb.184.16.4455-4465.2002). Statistical analysis: one-way ANOVA. *p* values: **** $p<0.0001$, ns = non-significant (compared to MH). Biological replicates: n=3. **C.** Impact of 200 µg/ml linezolid on *E. coli* WT growth after overnight culture in MH with or without 0.5% uridine. Results are shown as CFU/ml. Biological replicates: n=3. **D.** Inner membrane permeability was determined using propidium iodide (PI) in *E. coli* overexpressing CmtAB compared to the empty vector (p0) and *E. coli* WT grown with glucose or 0.5% uridine. Polymyxin was used as a membrane permeabilization control. Fisher's LSD test for statistical analysis. Biological replicates: n=3. **E.** Outer membrane permeability was monitored with nitrocefin treatment (OD 450nm) over 45 minutes in *E. coli* WT expressing bla, grown with glucose or 0.5% uridine. No significant difference was observed between conditions. Biological replicates: n=3.

Figure S7

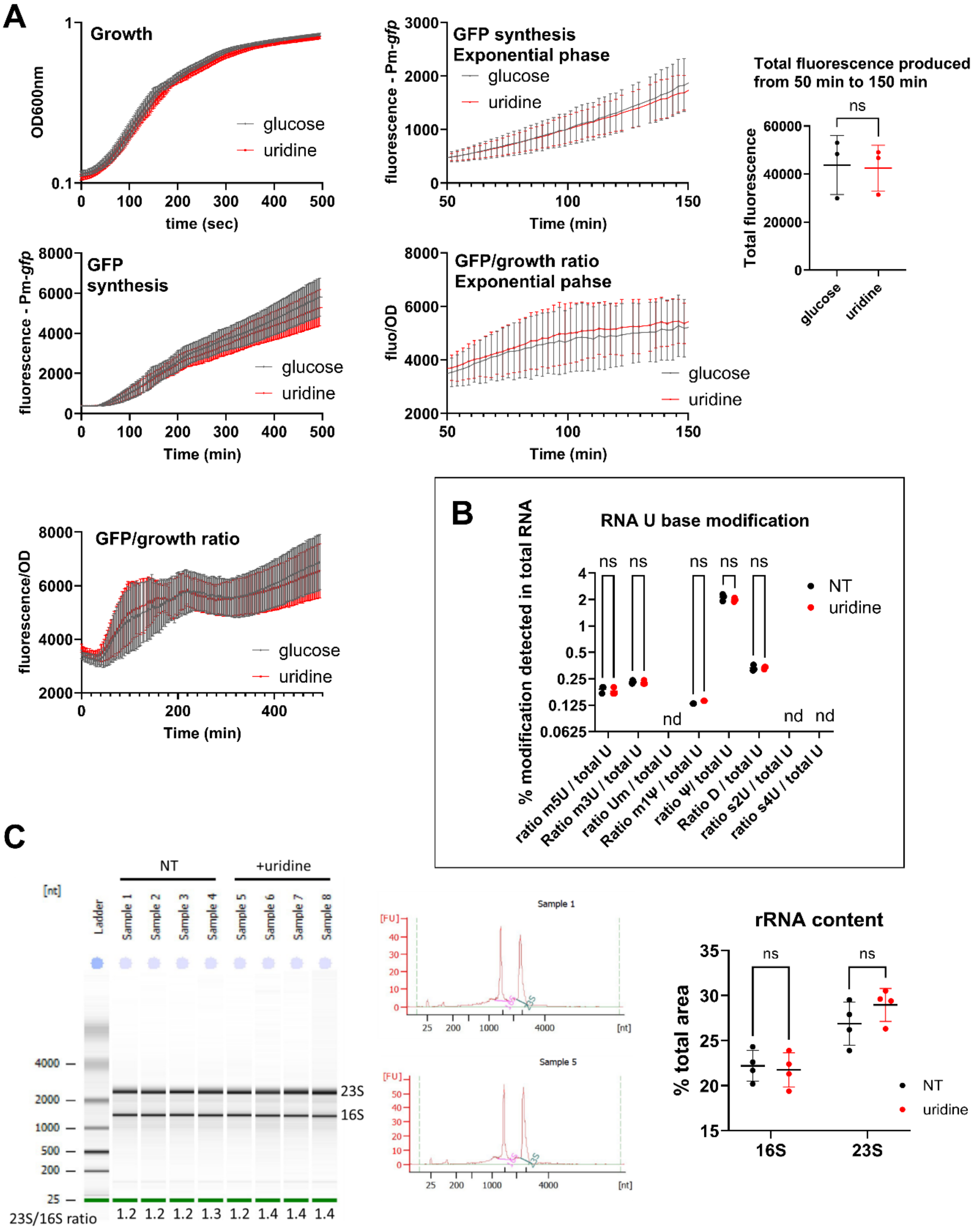

Figure S7. No effect of uridine on translation related phenotypes. A. *Translation speed assay:* GFP was cloned under the control of the inducible promoter Pm of pSEVA, and fluorescence was measured upon induction, as a function of time with glucose or uridine. **Left panel.** 1st graph: Growth was measured every 15 minutes over 500 minutes at OD 600nm in *E. coli* MG1655 expressing GFP in MH media with 0.5% glucose or uridine. 2nd graph: GFP fluorescence was measured in the same setup. 3rd graph: Ratio of fluorescence over growth for each time

point. **Middle panel:** The same graphs as the left panel, showing only the exponential phase (50-150 min). Total fluorescence produced during this phase was similar in both conditions, indicating that protein synthesis is not affected by uridine. Biological replicates: n=3. **B. RNA U base modification levels are not affected by uridine.** One parameter which could affect the ribosome is the levels of ribosomal RNA modifications. Using mass spectrometry, we measured whether U base modification levels change in the presence of uridine 0.5% supplementation: methyl-uridine (m5U, m3U, Um), pseudouridine (Ψ), methyl-pseudouridine (m1Ψ), dihydrouridine (D), thio-uridine (S2U, S4U). Indicated U modifications were quantified using mass spectrometry on RNA extracts from cultures grown in MH with and without uridine. Results are expressed as a percentage of total U. No difference in any modification levels has been detected in the presence or absence of uridine. Number of biological replicates for each experiment: n=3**. C. 16S and 23S rRNA contents are not affected by uridine.** We quantified the cellular 16S and 23S rRNA content using Bioanalyzer, comparing cultures grown with or without uridine 0.5%. No differences in the 16S/23S ratio or total RNA content were detected, indicating that uridine does not affect ribosome assembly or stability. Biological replicates: n=4.

Figure S8

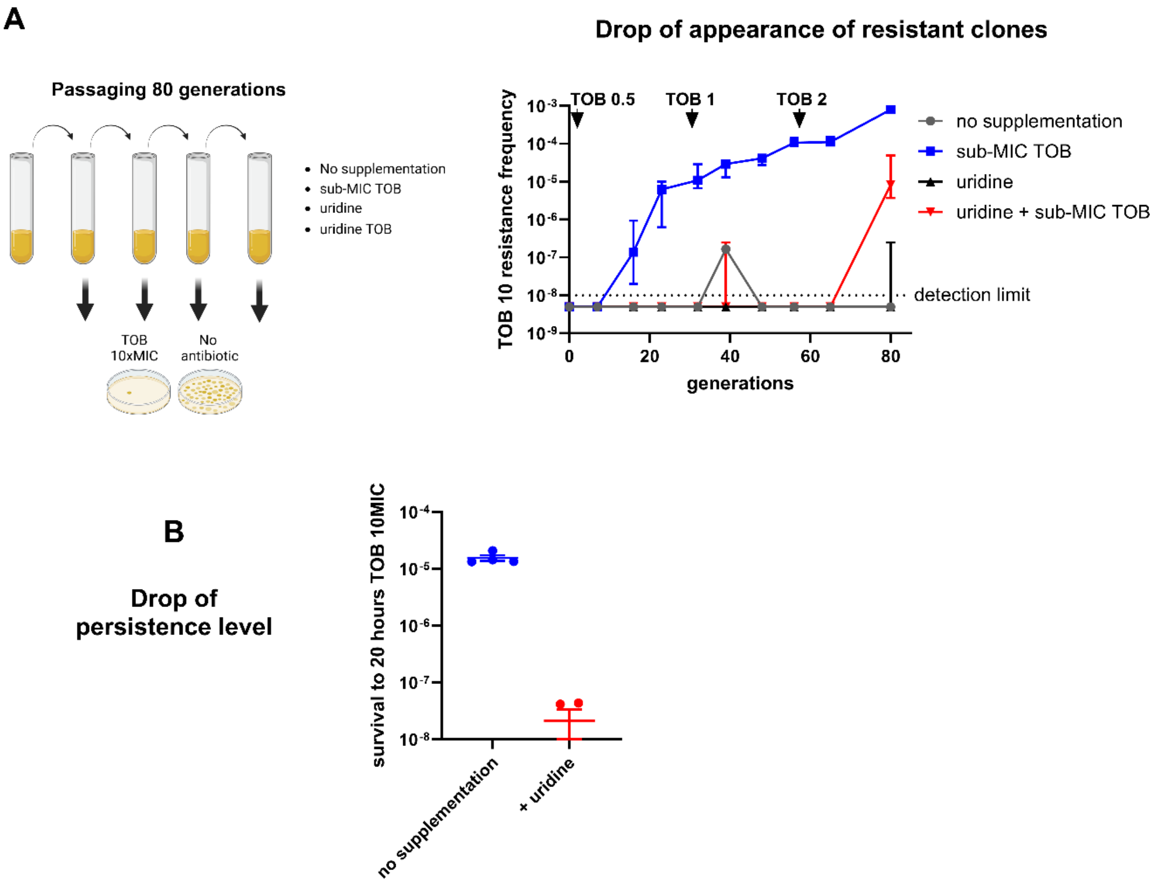

Figure S8: Uridine limits the selection of AG resistant mutants (A) and persistence (B). A. WT *E. coli* cultures were passaged for 80 generations in bactotryptone medium with or without sub-MIC tobramycin or uridine as indicated. Initial MIC of TOB in bactotryptone = 1 µg/ml. The initial sub-MIC TOB concentration was 0.5 µg/ml (50 % MIC) and gradually increased to 1 and 2 µg/ml as indicated. Overnight cultures were diluted 100X in 25 ml of medium containing 1% bactotryptone and 0.5 % NaCl, supplemented or not with uridine 0.5 %. +/- TOB. Each condition was tested in biological triplicates. Resistant clones were quantified by plating with a lethal concentration of tobramycin (10 µg/ml). **B.** Killing assay was performed as described in methods section in bactotryptone medium with and without uridine 0.5 % and plated after 20h TOB treatment (10 µg/ml), in triplicates. Survival was calculated by counting CFUs/ml after treatment divided by the initial number of CFUs/ml.

Figure S9

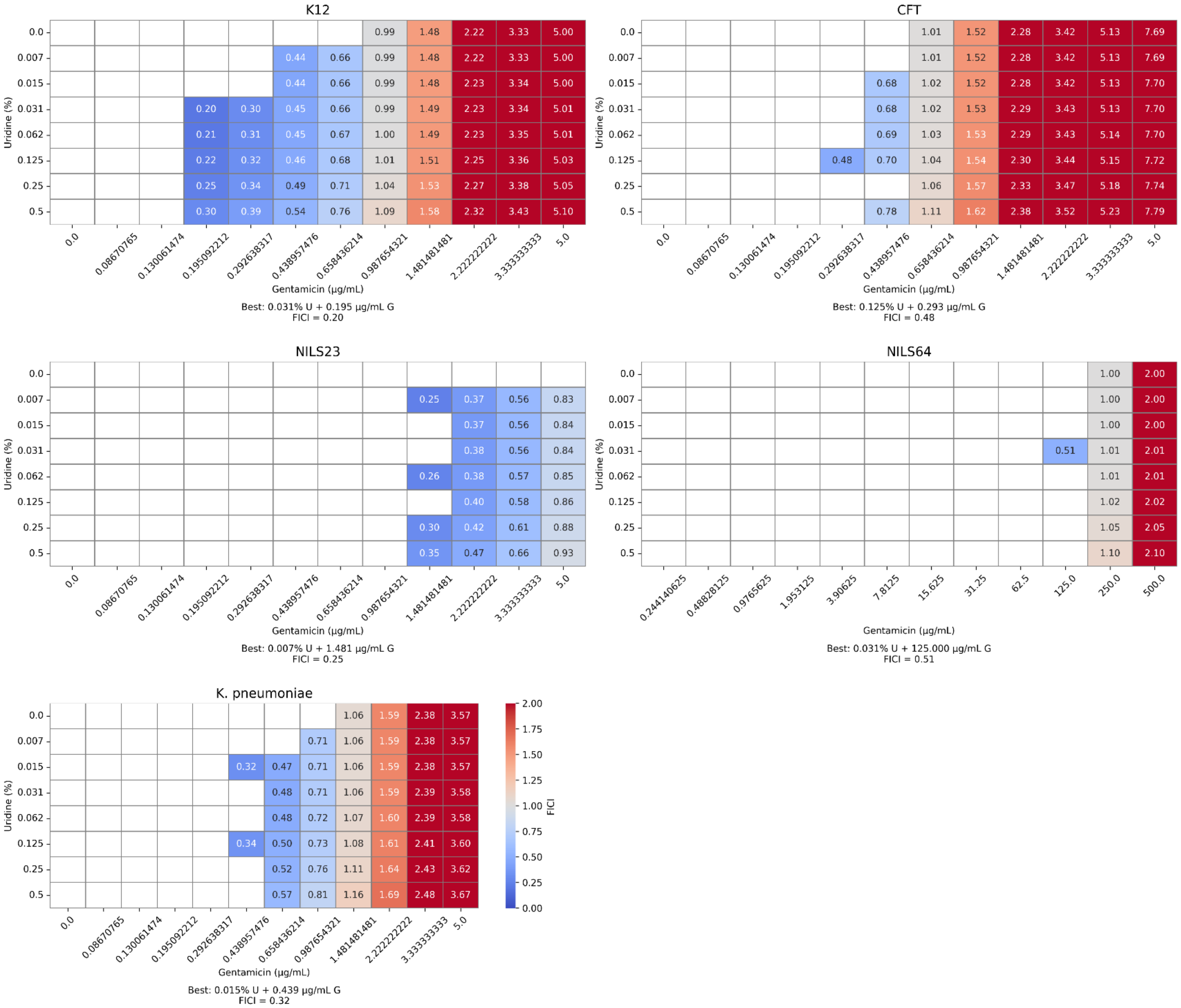

Figure S9 : Fractional Inhibitory Concentration Index (FICI) heatmap for the interaction between Uridine and Gentamicin in synthetic urine, for *E. coli* strains K12 (lab strain), CFT (gentamicin sensitive blood isolate), NILS23 (tolerant clinical isolate), NILS64 (resistant clinical isolate) and *K. pneumoniae*. The heatmaps display FICI values calculated from checkerboard assay data, using MICs of Uridine (5%) and Gentamicin when used individually. Only wells that resulted in complete bacterial inhibition (no visible growth) are included. Since uridine is not lethal alone, we attributed an arbitrary and low MIC value, 5%, for calculation purposes. FICI values were calculated using the formula:

FICI=(MICUridine in combo/MICUridine alone)+(MICGentamicin in combo/MICGentamicin alone)

Blue shading indicates **synergistic** interaction (FICI ≤ 0.5), while higher values represent **additive** (0.5 < FICI ≤ 1.0) or **indifferent** (>1.0) effects.

MICs (µg/ml) of gentamicin alone, determined in synthetic urine in checkerboard assays: For K12: 0.98 µg/mL; CFT073: 0.66; NILS23: 6; NILS64: 250; K. pneumoniae: 1.48.

FICIs. For K12, the lowest FICI observed was 0.21, indicating strong synergy at 0.031% Uridine + 0.2 µg/mL Gentamicin. For CFT, the lowest observed FICI was 0.47, indicating synergy at 0.125% Uridine + 0.3 µg/mL Gentamicin. Alternatively, additive effect is also observed with 0.031% Uridine + 0.43 µg/mL Gentamicin. For NILS23, the lowest FICI value observed was **0.26**, indicating synergy at 0.062% Uridine + 1.48 µg/mL Gentamicin. Alternatively, synergy is also observed with 0.031% Uridine + 2.22 µg/mL Gentamicin. For NILS64, the lowest FICI observed was 0.506, suggesting a near-synergistic/additive interaction at 0.031% Uridine + 125 µg/mL Gentamicin. For *K. pneumoniae*, the lowest FICI is 0.30, observed at 0.015% Uridine + 0.438 µg/mL Gentamicin, suggesting synergy. Alternatively, synergy is also observed with 0.031% Uridine + 0.65 µg/mL Gentamicin.

Table S1. MICs (µg/ml) in *E. coli* deletion, overexpression and clinical strains

**Gene deleted in *E. coli* MG1655 and associated substrate**	**Tobramycin MIC (µg/ml) determined by Etest**	**Medium**
WT	0.1	MH
lamB – Maltose	0.1	MH
malE – Maltose	0.12	MH
manY – Mannose	0.2	MH
fruA – Fructose	0.12	MH
bglH – ß-glucoside	0.1	MH
frwB – Fructose	0.1	MH
mngA – Fructose-like	0.12	MH
cmtA – Mannitol (cryptic) membrane component with *ubiI* E336* mutation	**0.4**	MH
cmtB – cytoplasmic component	0.1	MH
ptsG – Glucose	0.1	MH
mglA – Galactose	0.2	MH
chbC - Di-N-acetylchitobiose/Cellobiose	0.15	MH
nagE – Acetylglucosamine	0.12	MH
ypdG - Fructose-like	0.1	MH
ascF - Cellobiose/arbutine	0.1	MH
agaW - N-acetylgalactosamine	0.1	MH
sgcC -Pentose	0.1	MH
malX – Maltose	0.12	MH
mtlA – Mannitol	0.1	MH
treB – Trehalose	0.1	MH
frvB - Fructose-like	0.1	MH
agaC – Galactosamine	0.1	MH
srlE – Glucitol	0.12	MH
glvC – Arbutine	0.1	MH
ugpB – Glycerol	0.25	MH
alsB – Allose	0.15	MH
ytfQ – Galactofuranose	0.3	MH
crp	1.5	MH
udk	0.1	MH
udp	0.1	MH
BW25113 (Keio) WT	0.19	MH
BW25113 (Keio) *cmtA*	0.5	MH
BW25113 (Keio) *ubiI*	0.19-0.25	MH
**Overexpressed genes in *E. coli* MG1655**		
p0 (empty vector)	0.1	MH
lamB	0.1	MH
malEFG	0.08	MH
fruBKA	0.08	MH
frwBC	0.1	MH
mngA	0.08	MH
cmtAB	<0.064	MH
chbBCA	<0.064	MH
ascF	0.064	MH
malX	0.064	MH
frvAB	0.1	MH

srlEAB	0.064	MH
nupG	0.1	MH
nupC	0.1	MH
***E. coli* clinical strains**		
**NILS 9**	0.4	MH
**NILS 10**	0.5	MH
**NILS 24**	0.4	MH
**NILS 29**	0.4	MH
**NILS 31**	0.4	MH
**NILS 78**	0.25	MH
**886**	0.5	MH
**Ec019**	0.4	MH
**Ec068**	0.4	MH
**1236**	0.16	MH
**NILS 23**	2	MH
**NILS 46**	0.4	MH
**NILS 49**	0.5	MH
**1193#**	6	MH
**1195#**	0.5	MH
**932#**	14	MH
**NILS 64#**	7	MH
**1215#**	9.6	MH
**Ec120#**	14	MH
**CFT073**	0.3 (and MIC Gentamicin: 0.5)	MH
**UTI 89**	MIC Gentamicin: 0.5	MH
	**Gentamicin MIC (µg/ml) determined by microtiter broth dilution method**	**Medium**
MG1655	1.56	Tryptone
MG1655	**0.78**	**Tryptone + uridine**
MG1655	1.56	Tryptone + ribose
MG1655	1.56	Tryptone + glucose
p0 (empty vector)	1.56	Tryptone
p0 (empty vector)	**0.78**	**Tryptone + uridine**
p0 (empty vector)	1.56	Tryptone + ribose
p0 (empty vector)	1.56	Tryptone + glucose
p-cmtAB+	**0.78**	**Tryptone**
p-cmtAB+	**0.39**	**Tryptone + uridine**
p-cmtAB+	1.56	Tryptone + ribose
p-cmtAB+	1.56	Tryptone + glucose
MG1655	**<0.02**	**HPLM + uridine**
MG1655	<0.02	HPLM + ribose
MG1655	0.78	HPLM + glucose
p0 (empty vector)	**0.19**	**HPLM + uridine**
p0 (empty vector)	0.78	HPLM + ribose
p0 (empty vector)	0.78	HPLM + glucose
p-cmtAB+	**0.09**	**HPLM + uridine**
p-cmtAB+	0.39	HPLM + ribose
p-cmtAB+	1.56	HPLM + glucose
	**Ciprofloxacin MIC (µg/ml)**	**Medium**

	determined by microtiter broth dilution method	
MG1655	0.007	Tryptone
MG1655	0.004	Tryptone + uridine
p0 (empty vector)	0.007	Tryptone
p0 (empty vector)	0.007	Tryptone + uridine
p-cmtAB+	0.007	Tryptone
p-cmtAB+	0.007	Tryptone + uridine

# indicates strains with AG resistance genes. Sodium benzoate 1 mM was added to the culture for strains with plasmids to induce the expression of the transporter.

Table S2: Summary of effects of single transporter overexpressions in _E. coli_.

E. coli Transporter	Carbohydrate substrate (size)	Sensitizes to AGs	Sensitizes to CIP	Induced by uridine	Involved in AG potentiation by uridine
Inner membrane					
ABC transporters					
**_araFGH_**	L-arabinose (150 Da)	Could not be cloned, not tested			
**_*alsBACE_**	D-allose (180 Da)	Could not be cloned, not tested		Yes	
**_*mglBAC_**	D-galactose (180 Da)	**Yes**	**No**	**Yes**	**_mglABC_**
**_*malEFGH_**	Maltose (342 Da)	**Yes**	**mildly**	**Yes**	**_malEFG_**
**_*rbsDABC_**	D-ribose (150 Da)	Could not be cloned, not tested			
**_*ugpBAE_**	Glycerol (92 Da)	No	No	No	
**_xylEFGH_**	D-xylose (150 Da)	No	No	Not tested	
**_ytfQ_**	D-galactofuranose (180 Da)	**Yes**	**No**	Not tested	
PTS transporters					
**_agaCD_**	galactosamine	Could not be cloned, not tested			
**_ascFB_**	D-cellobiose/arbutine (342 Da)	**Yes**	**No**	**Yes**	**_ascFB_**
**_bglF_**	B-glucoside (cellobiose 342 Da)	**Yes**	**No**	**Yes**	**_bglF_**
**_chbBCA_**	Chitobiose (324 Da)	**Yes**	No	No	
**_cmtBA_**	Mannitol (182 Da)	**Yes**	**No**	**Yes**	**_cmtBA_**
**_*fruBKA_**	Fructose (180 Da)	**Yes**	**No**	**Yes**	**_fruBKA_**
**_frvAB_**	mannitol/fructose? (180 Da)	No	No	Not tested	
**_fryBC (ypdGH)_**	fructose? (180 Da)	**Yes**	**No**	**Yes**	**_fryBC (ypdGH)_**
**_*frwCB_**	Fructose (180 Da)	mildly	**No**	Yes	_frwCB_
**_*gatABCZ_**	Galactitol (182 Da)	**Yes**	Yes	No	
**_glvCB_**	Glucose/arbutine	**Yes**	**No**	Not tested	
**_*malXY_**	Maltose (342 Da)	**Yes**	**No**	**Yes**	**_malXY_**
**_*manXYZ_**	Mannose (180 Da)	**Yes**	**Yes**	**Yes**	**_manXYZ_**
**_mngA_**	mannosyl glycerate fructose? (180 Da)	**Yes**	No	No	
**_*mtlA_**	Mannitol (182 Da)	No	mildly	Not tested	
**_nagE_**	Acetylglucosamine (221 Da)	Could not be cloned, not tested			
**_ptsG_**	Glucose (180 Da)	No	No	Not tested	
**_*srlEAB_**	Sorbitol/glucitol (182 Da)	Yes	No	No	
**_*treBC_**	Trehalose (342 Da)	**Yes**	**Yes**	**Yes**	**_treBC_**
MFS transporters					
**_*fucP_**	Not tested because proton symporter				
**_galP_**	Galactose (180 Da)	No	mildly	Not tested	
**_*glpTQ_**	glycerol	**Yes**	**Yes**	**Yes**	**_glpTQ_**
**_lacY_**	Not tested because proton symporter				
Outer membrane					
**_bglH_**	B-glucoside (342 Da)	Yes	Yes	Yes	**_bglH_**
**_chiP_**	Chitotriose (486 Da)	No	mildly	Not tested	
**_lamB_**	Maltose (342 Da)	No	mildly	Not tested	
	**Total tested**	**27**			
	**Total AG sensitizing**	**19**			
	**Total AG sensitizing & induced by uridine**			**14**	

*: transporters downregulated by TOB sub-MIC

Table S3: Strains, plasmids and primers

**Strains**	**Number**	**Origin, construction or genotype**
Escherichia coli K12 subs. MG1655	C349	WT, Laboratory collection
Vibrio cholerae N16961	7805	WT, Laboratory collection
Pseudomonas aeruginosa PAO1	N065	WT, Laboratory collection
Klebsiella pneumoniae NTUH K2044	N105	WT, Liver abscess doi:10.1128/JB.00315-09
Acinetobacter baumannii ATCC 19606	I276	WT, Laboratory collection
**Deletions in *E. coli* MG1655**		
Δcrp	8542	Lab collection
Δcra	P940	P1 transduction from keio collection into MG1655
ΔcmtA	OO56	P1 transduction from keio collection into MG1655
ΔfruA	N812	P1 transduction from keio collection into MG1655
ΔchbC	OO66	P1 transduction from keio collection into MG1655
ΔlamB	N810	P1 transduction from keio collection into MG1655
ΔmalE	N811	P1 transduction from keio collection into MG1655
ΔfrwB	N813	P1 transduction from keio collection into MG1655
ΔfrvB	N814	P1 transduction from keio collection into MG1655
ΔptsG	N815	P1 transduction from keio collection into MG1655
ΔtreB	N816	P1 transduction from keio collection into MG1655
ΔmalX	N818	P1 transduction from keio collection into MG1655
ΔmtlA	N819	P1 transduction from keio collection into MG1655
ΔmglA	N820	P1 transduction from keio collection into MG1655
ΔmngA	N821	P1 transduction from keio collection into MG1655
ΔypdG	N822	P1 transduction from keio collection into MG1655
ΔsgcA	N823	P1 transduction from keio collection into MG1655
ΔsrlE	N824	P1 transduction from keio collection into MG1655
ΔascF	N825	P1 transduction from keio collection into MG1655
ΔagaW	OO64	P1 transduction from keio collection into MG1655
ΔbglF	OO67	P1 transduction from keio collection into MG1655
ΔsgcC	OO68	P1 transduction from keio collection into MG1655
ΔmanY	OO69	P1 transduction from keio collection into MG1655
ΔgalP	OO70	P1 transduction from keio collection into MG1655
ΔgatC	OO71	P1 transduction from keio collection into MG1655
ΔnagE	OO72	P1 transduction from keio collection into MG1655
ΔglvC	OO73	P1 transduction from keio collection into MG1655
Δudk	R060	P1 transduction from keio collection into MG1655
Δudp	R058	P1 transduction from keio collection into MG1655
ΔugpB		P1 transduction from keio collection into MG1655
ΔytfQ		P1 transduction from keio collection into MG1655
ΔalsB		P1 transduction from keio collection into MG1655
**Overexpression *E. coli* MG1655 unless otherwise indicated**		
pSEVA-238 (=p0)	O897	pM027 in MG1655 (lab collection)

pSEVA-238 *cmtAB*+ mannitol (cryptic)	P134	1860 bp PCR on gDNA using primers ML 290/291. Ligation into pSEVA-238 between EcorI and XbaI restriction sites.
pSEVA-238 *fruBKA*+ (fructose)	P314	3777 bp PCR on gDNA using primers ML 295/296. Ligation into pSEVA-238 between EcorI and XbaI restriction sites.
pSEVA-238 *malEFG*+ (maltose)	P132	3794 bp PCR on gDNA using primers ML 299/300. Ligation into pSEVA-238 between EcorI and XbaI restriction sites.
pSEVA-238 *manXYZ*+ (mannose)	P313	2699 bp PCR on gDNA using primers ML 301/302. Ligation into pSEVA-238 between EcorI and XbaI restriction sites.
pSEVA-238 *lamB*+ (maltose)	P311	1341 bp PCR on gDNA using primers ML 297/298. Ligation into pSEVA-238 between EcorI and XbaI restriction sites.
pSEVA-238 *frwBC*+ (fructose)	P715	1415 bp PCR on gDNA using primers ML 311/312. Ligation into pSEVA-238 between EcorI and XbaI restriction sites.
pSEVA-238 *mtlA*+ (mannitol)	P716	1911 bp PCR on gDNA using primers ML 303/304. Ligation into pSEVA-238 between EcorI and XbaI restriction sites.
pSEVA-238 *malX*+ (maltose)	P717	1592 bp PCR on gDNA using primers ML 315/316. Ligation into pSEVA-238 between KpnI and XbaI restriction sites.
pSEVA-238 *mngA*+ (fructose ?)	P718	1977 bp PCR on gDNA using primers ML 307/308. Ligation into pSEVA-238 between EcorI and XbaI restriction sites.
pSEVA-238 *glpTQ*+ (glycerol-3-phosphate)	P719	2440 bp PCR on gDNA using primers ML 317/318. Ligation into pSEVA-238 between EcorI and XbaI restriction sites.
pSEVA-238 *bglF*+ (β-glucoside)	P720	1879 bp PCR on gDNA using primers ML 323/324. Ligation into pSEVA-238 between EcorI and XbaI restriction sites.
pSEVA-238 *ypdGH*+ (fructose ?)	P721	1608 bp PCR on gDNA using primers ML 309/310. Ligation into pSEVA-238 between EcorI and XbaI restriction sites.
pSEVA-238 *bglH*+ (β-glucoside)	P722	1617 bp PCR on gDNA using primers ML 321/322. Ligation into pSEVA-238 between KpnI and XbaI restriction sites.
pSEVA-238 *treB*+ (trehalose)	P723	1422 bp PCR on gDNA using primers ML 313/314. Ligation into pSEVA-238 between KpnI and XbaI restriction sites.
pSEVA-238 *galP*+ (galactose)	Q144	1395 bp PCR on gDNA using primers ML 339/349. Ligation into pSEVA-238 between XbaI and PtsI restriction sites.
pSEVA-238 *gatABC*+ (galactitol)	Q145	2127 bp PCR on gDNA using primers ML 343/344. Ligation into pSEVA-238 between EcorI and XbaI restriction sites.
pSEVA-238 *chiP*+ (chitin)	Q146	1407 bp PCR on gDNA using primers ML 333/334. Ligation into pSEVA-238 between EcorI and XbaI restriction sites.
pSEVA-238 *chbCBA*+ (di-N-acetylchitobiose/cellobiose)	Q147	2165 bp PCR on gDNA using primers ML 331/332. Ligation into pSEVA-238 between EcorI and XbaI restriction sites.
pSEVA-238 *srlAEB*+ (arbutin)	Q148	1902 bp PCR on gDNA using primers ML 337/338. Ligation into pSEVA-238 between EcorI and XbaI restriction sites.
pSEVA-238 *acsF*+ (cellobiose/arbutin)	Q149	1458 bp PCR on gDNA using primers ML 335/336. Ligation into pSEVA-238 between EcorI and XbaI restriction sites.
pSEVA-238 *xylEFG*+ (D-xylose)	Q169	3770 bp PCR on gDNA using primers ML 325/326. Ligation into pSEVA-238 between EcorI and XbaI restriction sites.
pSEVA-238 *nupC*+ (cytidine, thymidine, uridine, adenosine)	Q872	1203 bp PCR on gDNA using primers ML 349/350. Ligation into pSEVA-238 between EcorI and XbaI restriction sites.
pSEVA-238 *nupG*+ (adenosine, guanine, thymidine, cytidine, uridine)	Q873	1257 bp PCR on gDNA using primers ML 351/352. Ligation into pSEVA-238 between EcorI and XbaI restriction sites.
pSEVA-238 *frvAB*+ (fructose ?)	Q168	1897 bp PCR on gDNA using primers ML 327/328. Ligation into pSEVA-238 between EcorI and XbaI restriction sites.
pSEVA-238 *ptsG*+ (glucose)	Q257	1434 bp PCR on gDNA using primers ML 345/346. Ligation into pSEVA-238 between EcorI and XbaI restriction sites.
MG1655 *ΔcmtA* + pSEVA238 *cmtA*+	Q322	Transformation of p134 in strain O056
MG1655 *ΔcmtA* + pSEVA238 p0	Q321	Transformation of empty plasmid in strain O056

pSEVA-238 *ytfQRTF*+ (galactofuranose)	X188	4657 bp PCR on gDNA using primers ML 540/541. PCR on pSEVA-238 using primers ML 542/543. Gibson assembly of the products.
pSEVA-238 *cmtB*+ (mannitol, intracellular component)	X189	485 bp PCR on gDNA using primers ML 552/553. PCR on pSEVA-238 using primers ML 554/555. Gibson assembly of the products.
pSEVA-238 *glvCB*+ (α-glucoside)	X190	1658 bp PCR on gDNA using primers ML 556/557. PCR on pSEVA-238 using primers ML 558/559. Gibson assembly of the products.
pSEVA-238 *mglBAC*+ (D-galactose-, D-galactoside)	X191	3639 bp PCR on gDNA using primers ML 564/565. PCR on pSEVA-238 using primers ML 566/567. Gibson assembly of the products.
pSEVA-238 *ugpBAEC*+ (sn-glycerol 3-phosphate)	X192	4257 bp PCR on gDNA using primers ML 560/561. PCR on pSEVA-238 using primers ML 562/563. Gibson assembly of the products.
pSEVA-238 *rbsDACB*+ (ribose)	X193	3793 bp PCR on gDNA using primers ML 568/569. PCR on pSEVA-238 using primers ML 570/571. Gibson assembly of the products.
**Overexpressions in *P. aeruginosa***		
pSEVA-238 (=p0)	P845	Transformation of pM027 into *P. aeruginosa*
pSEVA-238 *mtlFGK*+ of *P. aeruginosa* (maltose/mannitol)	P847	Transformation of pP828: 4315 bp PCR on gDNA using primers ML 365/366. Ligation into pSEVA-238 between BamHI and XbaI restriction sites.
pSEVA-238 *gtsB*+ of *P. aeruginosa* (mannose/glucose)	P848	Transformation of pP829: 933 bp PCR on gDNA using primers ML 355/356. Ligation into pSEVA-238 between EcoRI and XbaI restriction sites.
pSEVA-238 *oprB*+ of *P. aeruginosa* (glucose/mannitol/fructose /glycerol)	P849	Transformation of pP831: 1365 bp PCR on gDNA using primers ML 359/360. Ligation into pSEVA-238 between EcoRI and XbaI restriction sites.
pSEVA-238 PA2291 *oprB2* of *P. aeruginosa* (glucose/mannitol/fructose /glycerol)	R434	Transformation of pR433: 1359 bp PCR on gDNA using primers ML 414/415. Ligation into pSEVA-238 between EcoRI and XbaI restriction sites.
pSEVA-238 *fruAKI*+ of *P. aeruginosa* (fructose)	S298	Transformation of pR963: 5652 bp PCR on gDNA using primers ML 363/364. Ligation into pSEVA-238 between EcoRI and XbaI restriction sites. This study
**Overexpression in *A. baumannii***:		
pSEVA-238 (=p0)	R978	Transformation of pM027 into *A. baumannii*
pSEVA-238 *fruA*+ of *A. baumannii* (fructose)	R980	Transformation of pR962: 1959 bp PCR on gDNA using primers ML 431/432. Ligation into pSEVA-238 between EcoRI and XbaI restriction sites.
**GFP fusions in MG1655 unless otherwise indicated:**		
**pSC101**	O896	Lab collection
psc101 p*cmtA*-GFP	O537	pO501 in MG1655. PCR on gDNA using primers ML 252/253. Ligation into pTOPO-TA. Digestion with EcoRI and subcloning into psc101
MG1655 *Δcrp* + psc101 p*cmtA*-GFP	O538	pO501 in strain 8542
MG1655 *Δcra* psc101 p*cmtA*-GFP	Q115	pO501 in strain P940
MG1655 *ΔcmtA* + psc101 p*cmtA*-GFP	P315	pO501 in strain OO56
psc101-p*btuB*-GFP	R051	PCR on gDNA using primers ML 411/412. Ligation into pTOPO-TA. Digestion with EcoRI and subcloning into psc101.

psc101 p1*rrnB*-GFP	R921	pR692 in MG1655. Fruchard et al., 2022
psc101 p*fruA*-GFP	P115	PCR on gDNA using primers ML 261/262. Ligation into pTOPO-TA. Digestion with EcoRI and subcloning into psc101.
psc101 p*malE*-GFP	X210	PCR on gDNA using primers LH232/LH233. PCR on pSC101 with primers PSC101-F/PSC101-R. Gibson assembly.
psc101 p*ypdH*-GFP	X213	PCR on gDNA using primers LH230/LH231. PCR on pSC101 with primers PSC101-F/PSC101-R. Gibson assembly.
psc101 p*bglF*-GFP	X225	PCR on gDNA using primers LH258/LH259. PCR on pSC101 with primers PSC101-F/PSC101-R. Gibson assembly.
psc101 p*malX*-GFP	X207	PCR on gDNA using primers LH228/LH229. PCR on pSC101 with primers PSC101-F/PSC101-R. Gibson assembly.
psc101 p*asc*-GFP	X216	PCR on gDNA using primers LH251/LH252. PCR on pSC101 with primers PSC101-F/PSC101-R. Gibson assembly.
psc101 p*manX*-GFP	X228	PCR on gDNA using primers LH260/LH261. PCR on pSC101 with primers PSC101-F/PSC101-R. Gibson assembly.
psc101 p*gatZ*-GFP	X234	PCR on gDNA using primers LH265/LH266bis. PCR on pSC101 with primers PSC101-F/PSC101-R. Gibson assembly.
psc101 p*treB*-GFP	X220	PCR on gDNA using primers LH254/LH255. PCR on pSC101 with primers PSC101-F/PSC101-R. Gibson assembly.
psc101 p*glpT*-GFP	X231	PCR on gDNA using primers LH262/LH263. PCR on pSC101 with primers PSC101-F/PSC101-R. Gibson assembly.
psc101 p*frwC*-GFP	X222	PCR on gDNA using primers LH256/LH257. PCR on pSC101 with primers PSC101-F/PSC101-R. Gibson assembly.
psc101 p*srlE*-GFP	X406	PCR on gDNA using primers LH266/LH267. PCR on pSC101 with primers PSC101-F/PSC101-R. Gibson assembly.
psc101 p*mglB*-GFP	X407	PCR on gDNA using primers LH357/LH358. PCR on pSC101 with primers PSC101-F/PSC101-R. Gibson assembly.
psc101 p*alsB*-GFP	X409	PCR on gDNA using primers LH359/LH360. PCR on pSC101 with primers PSC101-F/PSC101-R. Gibson assembly.
psc101 p*ugp*-GFP	X412	PCR on gDNA using primers LH361/LH362. PCR on pSC101 with primers PSC101-F/PSC101-R. Gibson assembly.
psc101 p*glvC*-GFP	X415	PCR on gDNA using primers LH363/LH364. PCR on pSC101 with primers PSC101-F/PSC101-R. Gibson assembly.
psc101 p*bglH*-GFP	X418	PCR on gDNA using primers LH369/LH370. PCR on pSC101 with primers PSC101-F/PSC101-R. Gibson assembly.
**Suppressors growing on tobramycin + maltose after 20 hours of treatment (Figure 2F)**		
MG1655 big colonies	R796, R797 R798	fusA 1779T>G , rplL 122_127delTAGCTG
MG1655 small colony	R799	fusA 2015C>T, rplL 122_127delTAGCTG
MG1655 small colony	R800	fusA 2011C>T
**Clinical train number and stab**	Identified known Resistance genes	
886 (anal)	-	
1193 (skin)	blaCTX-M-14_1, blaTEM-1B_1, blaOXA-1_1, ant(3'')-Ia_1, aac(3)-IId_1, aadA5_1, sul1_2, dfrA17_1, aph(3')-Ia_1, mph(A)_1, mph(A)_2, tet(A)_4, catA1_1	
1195 (feces)	blaCTX-M-1_6, blaTEM-1B_1, aadA2_2, sul1_2, sul2_2, dfrA12_1, aph(3'')-Ib_5; aph(3')-Ia_1; aph(6)-Id_1, mph(A)_1; mph(A)_2, tet(A)_4	
1215 (skin)	ant(3'')-Ia_1, aac(6')-IIc_1, sul1_2, tet(A)_4	
1238 (feces)	ant(2'')-Ia_18; ant(3'')-Ia_1, sul1_2, sul2_2, aph(3'')-Ib_5; aph(6)-Id_1	
1236 (skin)	blaTEM-1B_1, blaSHV-2_2, aadA5_1, sul1_2, sul2_2, sul2_2	
Ec019 (anal)	blaCTX-M-14, mdfA_(1)	
Ec120 (anal)	mdfA_(1), ant(2'')-Ia_1, ant(3'')-Ia_1, sul1_5, sul2_2	

Ec068 (colostomy)	mdf(A)_1	
**Primer Number**	**Name**	**Sequence**
ML 252	FcmtA-5	TCAATTATGTAATATGCATCACG
ML 253	FcmtAGFP-3	AGTTCTTCTCCTTTACGCATAAATTATCCTTATTTTATTT
ML 261	FEcfruA-5	GCAATTAGGAAAAATGGC
ML 262	FEcfruA-3	GTTCTTCTCCTTTACGCATAGTTCTCCTCTCTTGCTG
ML 290	5SEVAcmtAecoRI	GCGGAATTCATGCGGCTTAGTGATTATTT
ML 291	3SEVAcmtAxbaI	GCGTCTAGATCAGTGTTTATGTTCGGCGG
ML 296	5SEVAfruBKAecoRI	CGCGGAATTCATGTTCCAGTTATCCGTACAGGAC
ML 297	3SEVAfruBKAxbaI	CGCGTCTAGATTACGCTGCTTTCGCTACTGC
ML 297	5SEVAlamBecoRI	CGCGGAATTCATGATGATTACTCTGCGCAAAC
ML 298	3SEVAlamBxbaI	CGCGTCTAGATTACCACCAGATTTCCATCTGG
ML 299	5SEVAmalEFGecoRI	CGCGGAATTCATGAAAATAAAAACAGGTGCACG
ML 300	3SEVAmalEFGxbaI	CGCGTCTAGATTAACCTTTCACACCACCTGCCG
ML 301	5SEVAmanXYZecoRI	CGCGGAATTCGTGACCATTGCTATTGTTATAGG
ML 302	3SEVAmanXYZxbaI	CGCGTCTAGATTACAGTCCCAGCAGGCCGC
ML 303	5SEVAmtlAecoRI	CGCGGAATTCATGAATAAGAAGGTGTTAACCCTGTC
ML 304	3SEVAmtlAxbaI	CGCGTCTAGATACTTACGACCTGCCAGCAGTTGCAC
ML 307	5SEVAmngAecoRI	CGCGGAATTCATGGTATTGTTTTATCGGGCAC
ML 308	3SEVAmngAxbaI	CGCGTCTAGATTATGGCATTACGCCATCAG
ML 309	5SEVAypdHGecoRI	CGCGGAATTCATGAGTAAGAAACTGATTGCC
ML 310	3SEVAypdHGxbaI	CGCGTCTAGATTACAGGCTATCGATTAACAATTTG
ML 311	5SEVAfrwBCecoRI	CGCGGAATTCATGAATGAGTTGGTGCAGATC
ML 312	3SEVAfrwBCxbaI	CGCGTCTAGATTAAGCGGTTTGCGCCAGGTG
ML 313	5SEVAtreBKpnI	CGCGGGTACCATGATGAGCAAAATAAACC
ML 314	3SEVAtreBxbaI	CGCGTCTAGATTAAACAATGTCCAGCGTGC
ML 315	5SEVAmalXKpnI	CGCGGGTACCATGACGGCGAAAACAGCACCG
ML 316	3SEVAmalXxbaI	CGCGTCTAGATTATGCCTGGACAGTATGCATCAGAC
ML 317	5SEVAglpTQecoRI	CGCGGAATTCATGTTGAGTATTTTTAAACCAG
ML 318	3SEVAglpTQXbaI	CGCGTCTAGATTACTCTTTATTAAGAAATTTTAC
ML 321	5SEVAbglHKpnI	CGCGGGTACCATGTTTAGACGAAATCTTATTAC
ML 322	3SEVAbglHxbaI	CGCGTCTAGATTACCACCAGATTTCAGCCTGGG
ML 323	5SEVAbglFecoRI	CGCGGAATTCATGACGGAGTTAGCCAGAAAAATAG
ML 324	3SEVAbglFxbaI	CGCGTCTAGATTAGCGAATGATGGATAACAGCG
ML 325	5SEVAxylFGHecoRI	GCGGAATTCATGAAAATAAAGAACATTCTAC
ML 326	3SEVAxylFGHxbaI	GCGTCTAGATCAAGAACGGCGTTTGGTTGC

ML 327	5SEVAfrvABecoR I	GCGGAATTCATGGCAGCTCTTACTGCAAGC
ML 328	3SEVAfrvABxbaI	GCGTCTAGAGACAAGCTCCTGTTGCGCGGCTTTC
ML 331	5SEVAchcBAeco RI	GCGGAATTCATGGAAAAGAAACACATTTATCTG
ML 332	3SEVAchCBAxba I	GCGTCTAGATTATGCCTTCAGTTTTTCATGAAGC
ML 333	5SEVAchiPecoRI	GCGGAATTCATGCGTACGTTTAGTGGCAAACG
ML 334	3SEVAchiPxbaI	GCGTCTAGATCAGAAGATGGTGAATGGTGCG
ML 335	5SEVAascFecoRI	GCGGAATTCATGGCCAAAAATTATGCGGCGCTG
ML 336	3SEVAscFxbaI	GCGTCTAGATCAATTAAGACTTACTTCTTTGG
ML 337	5SEVAsrlAEBeco RI	GCGGAATTCATGATAGAAACCATTACTCATGG
ML 338	3SEVAsrlAEBxba I	GCGTCTAGATTACTCCTTAACAGATTCAAACTTC
ML 339	5SEVAgalPxbaI	GCGTCTAGAATGCCTGACGCTAAAAAACAGG
ML 340	3SEVAgalPptsI	GCGCTGCAGTTAATCGTGAGCGCCTATTTCG
ML 341	5SEVAglvCBecoR I	GCGGAATTCATGCTCAGTCAAATTCAACGC
ML 342	3SEVAglvCBxbaI	GCGTCTAGATGCCTCCGTAATGGCAACATTTTCTG
ML 343	5SEVAgatABCec oRI	GCGGAATTCATGACTAACCTGTTTGTTCG
ML 344	3SEVAgatABCxb aI	GCGTCTAGATTATTCTGCGAGAACGACTTTCTC
ML 345	5SEVApstGecoRI	GCGGAATTCATGTTTAAGAATGCATTTGCTAACC
ML 346	3SEVAptsGxbaI	CGCGTCTAGATTAGTGGTTACGGATGTACTCATCC
ML 349	5SEVAnupCEcoR I	GCGGAATTCATGGACCGCGTCCTTCATTTTGTAC
ML 350	3SEVAnupCXbaI	CGCGTCTAGATTACAGCACCAGTGCTGCGATTGAC
ML 351	5SEVAnupGEcoR I	GCGGAATTCATGAATCTTAAGCTGCAGCTGAAAATC
ML 352	3SEVAnupGXbaI	CGCGTCTAGATTAGTGGCTAACCGTCTGTGTGCCTG
ML 355	5SEVAPagtsBEco rI	CGCGGAATTCATGGCGACCAATTCCCC
ML 356	3SEVAPagtsBxba I	CGCGTCTAGATCACGCATGGCGCTTGCC
ML 357	5SEVAPPalamBE corI	CGCGGAATTCATGAACAACCTGTTGTTG
ML 358	3SEVAPalamBxb aI	CGCGTCTAGATCATGCCGCCTCCAGGGCG
ML 359	5SEVAPaoprBEc orI	CGCGGAATTCATGTACAAGAACAAGAAAACC
ML 360	3SEVAPoprBxbaI	CGCGTCTAGATCAGAACACCGTCTGGATC
ML 363	5SEVAPafruopE orI	GCGGAATTCATGCTCGAACTCGATACCC
ML 364	3SEVAPafruopxb aI	GCGTCTAGAGTTATCCGCCGGTTCTCCGGAGT
ML 365	5SEVAVamalopB amHI	CGCGGGATCCATGAACGACTCGATCAAGGC
ML 366	3SEVAPamalopX baI	CGCGTCTAGATCAGGCCGCCTGTTGCAGTC
ML 411	FEcbtuB-5	GAGCTGACGCGCAGCGGTAAG
ML 412	FEcbtuB-3	GTTCTTCTCCTTTACGCATTGTAAAGCATCCACAATAG

ML 414	5SEVAPA2291ECorI	GCGGAATTCGTGAAATCCCATCTTCTCCG
ML 415	3SEVAPA2291xbaI	GCGTCTAGACTAGAACACCGTCTGGATCTTG
ML 431	5SEVAAbfruAecoRI	GCGGAATTCCGAATCCCTACTTCTGAGGTTC
ML 432	3SEVAAbfruAXbaI	GCGTCTAGACCTGAGCAAATGATGGCCCGTG
ML 536	nagE-for	GGCCGCGGCCGCGCGAATTCATGAATATTTTAGGTTTTTTC
ML 537	nagE-rev	GATCCCCGGGTACCGAGCTCTTACTTTTTGATTTCATAC
ML 538	SEVAnagE-for	GTATGAAATCAAAAAGTAAGAGCTCGGTACCCGGGGATC
ML 539	SEVAnagE-rev	GAAAAAACCTAAAATATTCATGAATTCGCGCGGCCGCGGCC
ML 540	Ytf-for	GCCGCGGCCGCGCGAATTCATGTGGAAACGCTTACTTATA
ML 541	Ytf-rev	GGATCCCCGGGTACCGAGCTCTTATTGCTGCGCAATGTTG
ML 542	SEVAYtf-for	CAACATTGCGCAGCAATAAGAGCTCGGTACCCGGGGATCC
ML 543	SEVAYtf-rev	TATAAGTAAGCGTTTCCACATGAATTCGCGCGGCCGCGGC
ML 544	Als-for	GGCCGCGGCCGCGCGAATTCATGAATAAATATCTGAAATATTTC
ML 545	Als-rev	GATCCCCGGGTACCGAGCTCTTACTTACTGATAAGACGG
ML 546	SEVAAls-for	CCGTCTTATCAGTAAGTAAGAGCTCGGTACCCGGGGATC
ML 547	SEVAAls-rev	GAAATATTTCAGATATTTATTCATGAATTCGCGCGGCCGCGGCC
ML 548	cmtA-for	GGCCGCGGCCGCGCGAATTCATGGAAAACAAGTCTGCTCG
ML 549	cmtA-rev	GATCCCCGGGTACCGAGCTCTCAGTGTTTATGTTCGGCGG
ML 550	SEVAcmtA-for	CCGCCGAACATAAACACTGAGAGCTCGGTACCCGGGGATC
ML 551	SEVAcmtA-rev	CGAGCAGACTTGTTTTCCATGAATTCGCGCGGCCGCGGCC
ML 552	cmtB-for	GGCCGCGGCCGCGCGAATTCATGCGGCTTAGTGATTATTTTC
ML 553	cmtB-rev	GGATCCCCGGGTACCGAGCTCTTATCCGCGGCTGATAATG
ML 554	SEVAcmtB-for	CATTATCAGCCGCGGATAAGAGCTCGGTACCCGGGGATCC
ML 555	SEVAcmtB-rev	GAAAATAATCACTAAGCCGCATGAATTCGCGCGGCCGCGGCC
ML 556	glvCB-for	GGCCGCGGCCGCGCGAATTCATGCTCAGTCAAATTCAACG
ML 557	glvCB-rev	GGATCCCCGGGTACCGAGCTCTTATACTGCCTCCGTAATGGC
ML 558	SEVAglv-for	GCCATTACGGAGGCAGTATAAGAGCTCGGTACCCGGGGATCC
ML 559	SEVAglv-rev	CGTTGAATTTGACTGAGCATGAATTCGCGCGGCCGCGGCC
ML 560	Ugp-for	GGCCGCGGCCGCGCGAATTCATGAAACCGTTACATTATAC
ML 561	Ugp-rev	GGATCCCCGGGTACCGAGCTCTCATACTCGTTGTCCTGTTTC
ML 562	SEVAugp-for	GAAACAGGACAACGAGTATGAGAGCTCGGTACCCGGGGATCC
ML 563	SEVAugp-rev	GTATAATGTAACGGTTTCATGAATTCGCGCGGCCGCGGCC
ML 564	Mgl-for	GGCCGCGGCCGCGCGAATTCATGAATAAGAAGGTGTTAACC
ML 565	Mgl-rev	GATCCCCGGGTACCGAGCTCTCATTTCTTACGCGCGTATTTC
ML 566	SEVAMgl-for	GAAATACGCGCGTAAGAAATGAGAGCTCGGTACCCGGGGATC
ML 567	SEVAMgl-rev	GGTTAACACCTTCTTATTCATGAATTCGCGCGGCCGCGGCC
ML 568	Rbs-for	GGCCGCGGCCGCGCGAATTCATGAAAAAAGGCACCGTTC
ML 569	Rbs-rev	GGATCCCCGGGTACCGAGCTCCTACTGCTTAACAACCAGTTTC
ML 570	SEVArbs-for	GAAACTGGTTGTTAAGCAGTAGGAGCTCGGTACCCGGGGATCC
ML 571	SEVArbs-rev	GAACGGTGCCTTTTTTCATGAATTCGCGCGGCCGCGGCC
LH228	malX-for	CAACAGGCTTACCCGTCTTACCCGGGGATCCatcttatacccgctattatcgttgc
LH229	malX-rev	GAAAAGTTCTTCTCCTTTACGCATaaaacgactcctcgtagagaac

LH230	ypdH-for	CAACAGGCTTACCCGTCTTACCCGGGGATCCgtcacacttccgtacagcgg
LH231	ypdh-rev	GAAAAGTTCTTCTCCTTTACGCATcgttaattcctcgttcagtga
LH232	malE-for	CAACAGGCTTACCCGTCTTACCCGGGGATCCtctccacattaccgccaatt
LH233	malE-rev	GAAAAGTTCTTCTCCTTTACGCATaatctatggtccttgttggtg
LH234	gfp	ATGCGTAAAGGAGAAGAACTTTTC
LH235	gfp	GTACCGAGCTCGAATTCCGACAAGCCAAGCTTTTATTTGTATAGTTCATCCATGCCATG
LH251	ascF-for	CAACAGGCTTACCCGTCTTACCCGGGGATCCattcaggtgaccggtttcac
LH252	ascF-rev	GAAAAGTTCTTCTCCTTTACGCATcggattttatcctgttatcagtagg
LH254	treB-for	CAACAGGCTTACCCGTCTTACCCGGGGATCCaccctgtcctgatcgtttcc
LH255	treB-rev	GAAAAGTTCTTCTCCTTTACGCATaaagccccatggcagatgac
LH256	frwC-for	CAACAGGCTTACCCGTCTTACCCGGGGATCCgcaagttatgaggcggatcg
LH257	frwC-rev	GAAAAGTTCTTCTCCTTTACGCATaagctgctcccgggcttg
LH258	bglF-for	CAACAGGCTTACCCGTCTTACCCGGGGATCCcaacgtaaaatttcacccgc
LH259	bglF-rev	GAAAAGTTCTTCTCCTTTACGCATaacttgccctctaccgctttg
LH260	manXY-for	CAACAGGCTTACCCGTCTTACCCGGGGATCCgctaatcgaaagttaaattacggatcttc
LH261	manXY-rev	GAAAAGTTCTTCTCCTTTACGCATttgctacctcctttattatcgttaacacc
LH262	glpT-for	CAACAGGCTTACCCGTCTTACCCGGGGATCCcgtgatttcatgcgtcattttgaac
LH263	glpT-rev	GAAAAGTTCTTCTCCTTTACGCATtgatagcctccgtggcccgt
LH265	gatZ-for	CAACAGGCTTACCCGTCTTACCCGGGGATCCccatcaaactggggatatgc
LH265	gatZ-rev	GAAAAGTTCTTCTCCTTTACGCATgtttttttccttgttaaatggcaggtgc
LH266	srlA-for	CAACAGGCTTACCCGTCTTACCCGGGGATCCctcaactcattcccctcgct
LH267	srlA-rev	GAAAAGTTCTTCTCCTTTACGCATtgttctctccttcaggatttattg
LH357	mlgB-for	CAACAGGCTTACCCGTCTTACCCGGGGATCCaattaaagccgttttctggagcg
LH358	mlgB-rev	GAAAAGTTCTTCTCCTTTACGCATggtatctccggtttttcttatgcagg
LH359	alsB-for	CAACAGGCTTACCCGTCTTACCCGGGGATCCctgtcgcccagctcaacatt
LH360	alsB-rev	GAAAAGTTCTTCTCCTTTACGCATaataatgttctcacggtaaggggac
LH361	ugpB-for	CAACAGGCTTACCCGTCTTACCCGGGGATCCacgccgtcaccgccttg
LH362	ugpB-rev	GAAAAGTTCTTCTCCTTTACGCATcgtttatctctcttgttgtaccgaatg
LH363	glvC-for	CAACAGGCTTACCCGTCTTACCCGGGGATCCtctctaccgcccaacgaaaag
LH364	glvC-rev	GAAAAGTTCTTCTCCTTTACGCATttccacatcctcttttctcaattctg
LH369	bglH-for	CAACAGGCTTACCCGTCTTACCCGGGGATCCcaagaaaagcttcggatggtatgc
LH370	bglH-rev	GAAAAGTTCTTCTCCTTTACGCATaatatccctttatggtgcaaagaaag
